# Additive Anti-Tumor Effects of Lovastatin and Everolimus In Vitro through Simultaneous Inhibition of Signaling Pathways

**DOI:** 10.1371/journal.pone.0143830

**Published:** 2015-12-04

**Authors:** Svenja Nölting, Julian Maurer, Gerald Spöttl, Elke Tatjana Aristizabal Prada, Clemens Reuther, Karen Young, Márta Korbonits, Burkhard Göke, Ashley Grossman, Christoph J. Auernhammer

**Affiliations:** 1 Department of Internal Medicine II, Campus Grosshadern, University-Hospital, Ludwig-Maximilians-University of Munich, Munich, Germany; 2 Department of Endocrinology, William Harvey Research Institute, Barts and the London School of Medicine, Queen Mary University of London, London, United Kingdom; 3 University-Hospital Hamburg-Eppendorf, Hamburg, Germany; 4 Department of Endocrinology, Oxford Centre for Diabetes, Endocrinology and Metabolism, Churchill Hospital, University of Oxford, Oxford, United Kingdom; Hunter College of The City University of New York, UNITED STATES

## Abstract

**Background:**

The mTORC1-inhibitor everolimus shows limited efficacy in treating patients with gastro-entero-pancreatic or pulmonary neuroendocrine tumors (NETs), and poor outcome in patients with malignant pheochromocytoma or hepatic carcinoma. We speculated that any effect may be enhanced by antogonising other signaling pathways.

**Methods:**

Therefore, we tested the effect of lovastatin—known to inhibit both ERK and AKT signaling—and everolimus, separately and in combination, on cell viability and signaling pathways in human midgut (GOT), pancreatic (BON1), and pulmonary (H727) NET, hepatocellular carcinoma (HepG2, Huh7), and mouse pheochromocytoma (MPC, MTT) cell lines.

**Results:**

Lovastatin and everolimus separately significantly reduced cell viability in H727, HepG2, Huh7, MPC and MTT cells at clinically relevant doses (P ≤ 0.05). However, high doses of lovastatin were necessary to affect GOT or BON1 cell viability. Clinically relevant doses of both drugs showed additive anti-tumor effects in H727, HepG2, Huh7, MPC and MTT cells (P ≤ 0.05), but not in BON1 or GOT cells. In all cell lines investigated, lovastatin inhibited EGFR and AKT signaling. Subsequently, combination treatment more strongly inhibited EGFR and AKT signaling than everolimus alone, or at least attenuated everolimus-induced EGFR or AKT activation. Vice versa, everolimus constantly decreased pp70S6K and combination treatment more strongly decreased pp70S6K than lovastatin alone, or attenuated lovastatin-induced p70S6K activation: in BON1 cells lovastatin-induced EGFR inhibition was least pronounced, possibly explaining the low efficacy and consequent absent additive effect.

**Conclusion:**

In summary, clinically relevant doses of lovastatin and everolimus were effective separately and showed additive effects in 5 out of 7 cell lines. Our findings emphasize the importance of targeting several interacting signaling pathways simultaneously when attempting to attenuate tumor growth. However, the variable reactions of the different cell lines highlight the necessity to understand the unique molecular aberrations in any tumor. Nevertheless, this combination seems worthy of being tested *in vivo*.

## Introduction

There are currently no generally effective and sustained systemic therapies for metastatic pheochromocytoma [1, 2] or neuroendocrine tumors (NETs) that mainly arise from the gastro-entero-pancreatic system and the lung [3–5]. The mTORC1-inhibitor everolimus has already been approved for the therapy of pancreatic NETs [6], and has shown therapeutic effects in midgut NETs [7]. Although mTORC1 inhibition by everolimus has proven to be a promising approach in NET therapy [6–8], the rapid development of drug resistance may be partly due to compensatory PI3K/AKT activation, or the induction of other signaling pathways [9–11].

The lipid lowering HMG-CoA-reductase-inhibitor lovastatin has been shown to have anti-tumor potential in many different cell lines, in animal models, and in clinical studies evaluating the effect of prior statin use on the incidence of different types of cancer, as recently reviewed [12]. A large retrospective study reported that statin use before cancer diagnosis resulted in a significant (15%) reduction in all-cause and cancer-related mortality [13]. Recent clinical studies have identified a reduced risk of prostate, esophageal, gastric, colorectal, hepatocellular, inflammatory breast, ovarian, endometrial, and renal cancer and glioma among statin users, although there has been reported an increased risk of bladder and intralobular or intraductal breast cancer [12]. Interestingly, a significantly lower risk of liver cancer has been reported in patients with pre-existing liver disease or diabetes who have been taking statins, suggesting a benefit in terms of chemoprevention in persons at elevated risk of liver cancer [14, 15].

**Fig 1:**
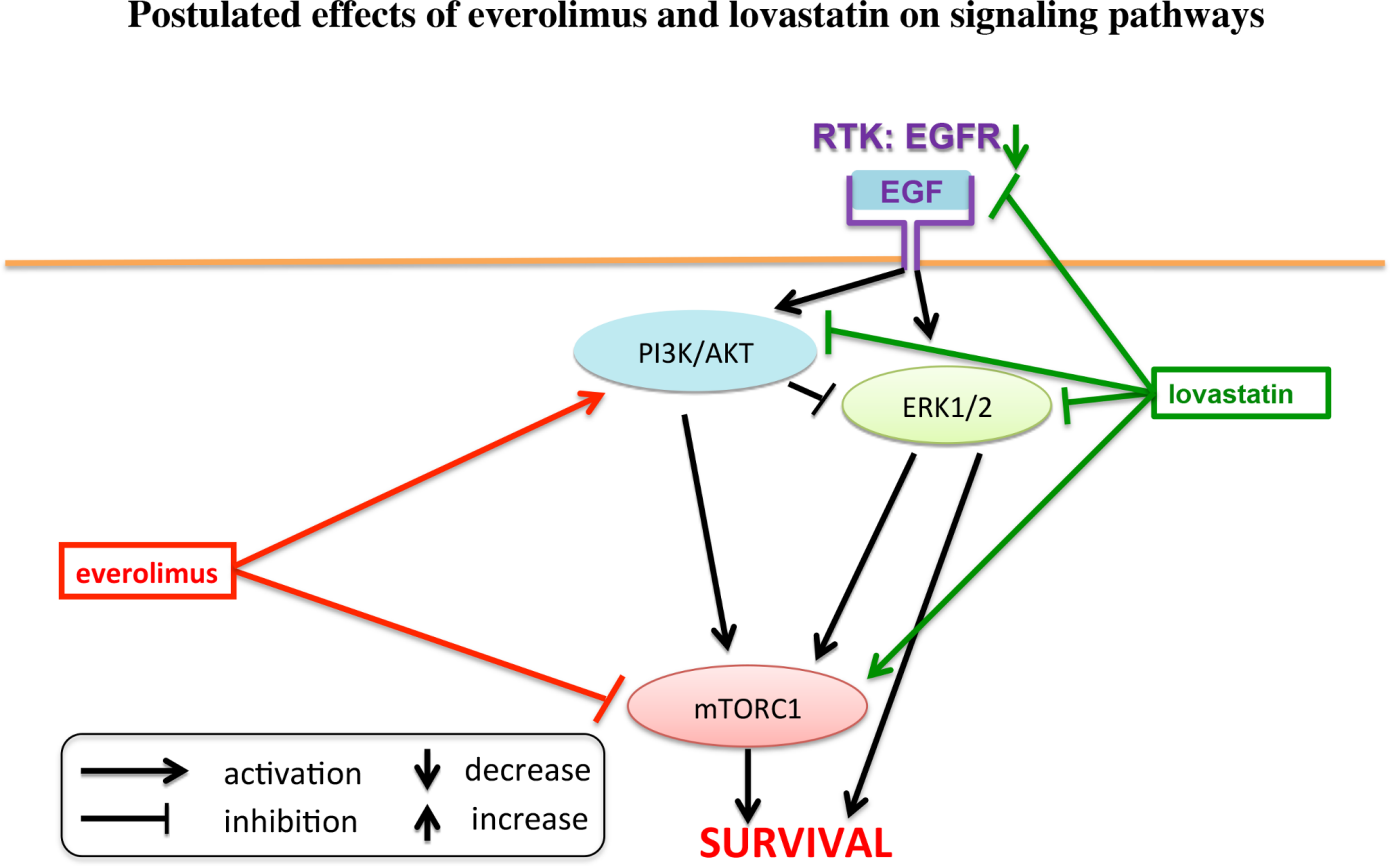
Postulated effects of everolimus and lovastatin on signaling pathways: lovastatin has been described to inhibit EGFR, AKT and ERK signaling, but has been found to increase mTORC1/p70S6K signaling; everolimus is known to inhibit mTORC1, but to increase AKT signaling.

As a possible mechanism of action in terms of an anti-oncogenic effect, statins have been reported to inhibit vascular endothelial growth factor receptor (VEGFR) and epidermal growth factor receptor (EGFR) signaling, to enhance the effect of receptor-tyrosine-kinase-inhibitors such as gefitinib and sorafenib [16–21], and to overcome EGFR-tyrosine-kinase-inhibitor resistance in non-small cell lung cancer (NSCLC) cells and thus improve progression free survival of NSCLC patients treated with EGFR-tyrosine-kinase-inhibitors [22, 23]. In two mouse pheochromocytoma cell lines (MPC and MTT), lovastatin was found to have anti-tumor potential and inhibit both AKT and ERK signaling, but to increase pp70S6K [24, 25]. Lovastatin also demonstrated a chemopreventive effect *in vivo* in a pheochromocytoma (MTT cell) allograft mouse model [26]. Thus, statins may have anti-tumor potential, particularly in combination with other chemotherapeutics or targeted therapies [16–23, 27–31], and may even show chemopreventive effects in certain contexts. Fig 1 schematically shows the postulated molecular effects of everolimus and lovastatin.

Therefore, in this study we investigated the following two hypotheses:

1. Lovastatin and everolimus would separately significantly reduce cell viability at clinically relevant doses in human pancreatic (BON1), midgut (GOT) and pulmonary (H727) NET cells, two mouse pheochromocytoma cell lines (MPC and MTT), and two human liver cancer cell lines (Huh7 and HepG2).
2. Both drugs would have an additive inhibitory effect at clinically relevant doses on cell viability of BON1, GOT, H727, MPC, MTT, Huh7 and HepG2 cells.

We tested two non-endocrine hepatic cell lines to assess if any effects were specific to NETs as opposed to cancers in general. We further explored the changes in signaling pathways which may mediate their anti-tumor effects. In summary, the primary hypothesis of significant reduction of cell viability by each drug separately and the secondary hypothesis of an additive effect of both drugs at clinically relevant doses was found to apply in 5 out of 7 cell lines. Neither of the two hypotheses applied to either BON1 or GOT cells, emphasizing the importance of considering the individual molecular aberrations in any tumor.

## Materials and Methods

### Reagents

Everolimus (07741 FLUKA) and lovastatin (M2147 SIGMA) were purchased from Sigma, St. Louis, MO, USA. For cell culture work, drugs were diluted in dimethyl-sulfoxide (DMSO, 10 mM stock solution; Sigma, D8418). DMSO was used at the appropriate dilution as control and found to be equivalent to the blank control up to concentrations of 0.4% DMSO (equivalent to 40 μM drug concentration) in the MTS assay and western blots. Dulbecco`s Modified Eagle medium–Nutrient Mixture F-12, 1:1 (DMEM/F12) media and penicillin/streptomycin were acquired from Gibco/Invitrogen (Karlsruhe, Germany), Trypsin-EDTA (10x) from PAA Laboratories (Cölbe, Deutschland), phosphate-buffered saline (PBS) and RPMI-Medium (with L-Glutamine, NaCO_3_) were purchased from Sigma. Fetal bovine serum (FBS) and amphotericin B were received from Biochrom (Berlin, Germany).

### Cell culture

All human neuroendocrine cell lines were received and cultured, as recently described [32]. Pancreatic neuroendocrine BON1 tumor cells [33] (kindly provided by Prof. R. Göke, Marburg, Germany) were grown in DMEM/F12 (1:1) supplemented with 10% FBS, 1% penicillin/streptomycin and 0.4% amphotericin B. Human midgut carcinoid GOT1 cells [34] (kindly provided by Prof. O. Nilsson, Sahlgrenska University Hospital Göteborg, Sweden) and human broncho-pulmonary neuroendocrine NCI-H727 tumor cells [35] (purchased from ATCC, Manassas, VA) were grown in RPMI medium supplemented with 10% FBS, 1% penicillin/streptomycin and 0.4% amphotericin B. Two mouse pheochromocytoma cell lines, MPC 4/30PRR mouse pheochromocytoma cells (MPC) [36] and mouse tumor tissue-derived (MTT; more aggressive) [37] were kindly provided by Dr Karel Pacak (NIH,Bethesda, MD, USA) and cultured as recently described [24]. Briefly, MPC and MTT cells were grown in medium containing 10% FBS and 1% penicillin/streptomycin. Both cell lines have been generated from heterozygous Nf1 knockout mice [36, 37]. Hepatocellular carcinoma cell lines HepG2 (purchased from ATCC, Manassas) and Huh7 (acquired from JCRB Cell Bank, Osaka, Japan) were cultured as described above for NCI-H727. The cells were mycoplasma free and incubated at 37°C in 5% CO_2_.

### Assessment of cell viability

Cells were counted by an automated cell counter (Countess, Invitrogen, Germany), seeded into 96-well plates at densities of 1500–3000 (BON1), 30000–50000 (GOT), 2000–4000 (NCI-H727, HepG2, Huh7), 20000 (MPC, MTT) cells per well and grown for 24h. Afterwards, cells were treated with various concentrations of lovastatin, 10 nM everolimus, or a combination of both drugs for the indicated time intervals in 10% FBS and 1% penicillin/streptomycin medium. Combination treated cells were always pre-treated with lovastatin for 24h and afterwards treated with the combination of both drugs for 48h or 120h. For comparability reasons, the single drugs were incubated for the same time as in combination (lovastatin for 72h or 144h, everolimus for 48h or 120h). Metabolic activity was assessed by “Cell Titer 96 Aqueous One Solution” cell viability assay (Promega, Madison, WI, USA) according to the manufacturer`s instructions after different incubation periods. Following 3h of incubation with Cell Titer 96 solution, the absorbance at 490 nm was recorded using an ELISA plate reader (Orion II, Berthold Detection Systems, Pforzheim, Germany).

### Protein Extraction and Western Blotting

For Western blot experiments, 4x10^5^ cells (BON1) or 5x10^5^ cells (NCI-H727, HepG2, Huh7) were seeded in 6-well plates and grown for 24h in complete medium. Afterwards the medium was replaced by fresh 10% FBS medium and cells were incubated with 20 μM lovastatin, 10 nM everolimus, or the combination of 10 nM everolimus and 20 μM lovastatin. For combination treatment, cells were pretreated for 24h with 20 μM lovastatin before 10 nM everolimus was added, and the combination of both drugs was incubated for another 48h. The cells were subsequently washed twice in cold PBS on ice and lysed in 200 μl lysis buffer (M-PER ® Mammalian Protein Extraction Reagent containing HALT protease & phosphatase inhibitor cocktail, Thermo Scientific, Rockford, USA). Lysates were centrifuged at 13,000 rpm for 10 min. Supernatants were adjusted to the same protein concentration (30–50 μg/50μl) (Rotiquant Universal, Carl Roth, Karlsruhe, Germany). Equal amounts of protein were denatured in Sodium dodecyl sulfate (SDS) sample buffer (0.25% Tris HCL, 40% glycerol, 2% SDS, 1% dithiothreitol, bromophenol blue, pH 8.8), separated on a SDS polyacrylamide gel and electrotransferred for 60 min onto PVDF membranes (Immobilone; Millipore, Eschborn, Germany) using a semi-dry Western-blot technique. After blocking in 2% skimmed milk powder, the membranes were incubated overnight at 4°C in appropriate dilutions of primary antibodies against pAKT (Ser473) (#4060), AKT (#2920), pERK (Thr202/Tyr204) 1/2 (#4370), pp70S6K (Thr389) (#9234), p70S6K (#9202), pEGFR (Tyr1068) (#3777), EGFR (#4267) (all from Cell Signaling, Danvers, MA), Actin (A5441) (Sigma, St. Louis, USA), ERK 1/2 (06–182) (Merck-Millipore, Darmstadt, Germany). After washing in TBS, the membranes were incubated with a peroxidase-conjugated secondary antibody (1:25000) for 2h. The blots were washed and immersed in the chemiluminescent substrate SuperSignal West Dura (Thermo Scientific, Rockford, USA) and images were taken with an ECL Chemocam Imager (INTAS, Göttingen, Germany). Optical density of the approximately sized bands was quantified using ImageJ 1.440 software (Wayne Rasband, National Institute of Health, USA).

Band intensities were quantified from at least 3 (in two cases), mostly 4–5 independent experiments for each cell line and protein, and are described as the mean percentage relative to the untreated control (100%). We have referred the phospho-protein expression to the control to adequately assess the extent of activation or inhibition of signaling pathways after drug treatment and compare it between the different cell lines.

### Statistical analysis

All results are shown as mean ± standard error of the mean (SEM) of at least three independently performed experiments. Each single cell viability experiment consisted of at least 6 samples per drug concentration and incubation period. The normality of the distribution of our data was analyzed via the Kolmogorov-Smirnov-Test and homogeneity of variances was determined by the Levene test. The Kolmogorov-Smirnov-Test suggested a deviation from a normal distribution while the Levene test showed absence of homogeneity of variances. Thus, for all data sets, the multiple-comparison Kruskal-Wallis-Test was implemented as a non-parametric alternative to the one way ANOVA. If the overall comparison test showed a significant difference, groups were compared pairwise with the Mann-Whitney test. Statistical significance was defined at P ≤ 0.05. For statistical analysis the program SPSS (version 13.0 for Windows, SPSS Inc (2005), Chicago, USA) was used. Cell viability and optical density of the approximately-sized bands of each Western blot are expressed as mean percentage relative to the untreated control (100%). Due to the exploratory character of this work, all P values have to be interpreted descriptively. An adjustment of P values was not performed.

## Results

### Cell viability

**Fig 2:**
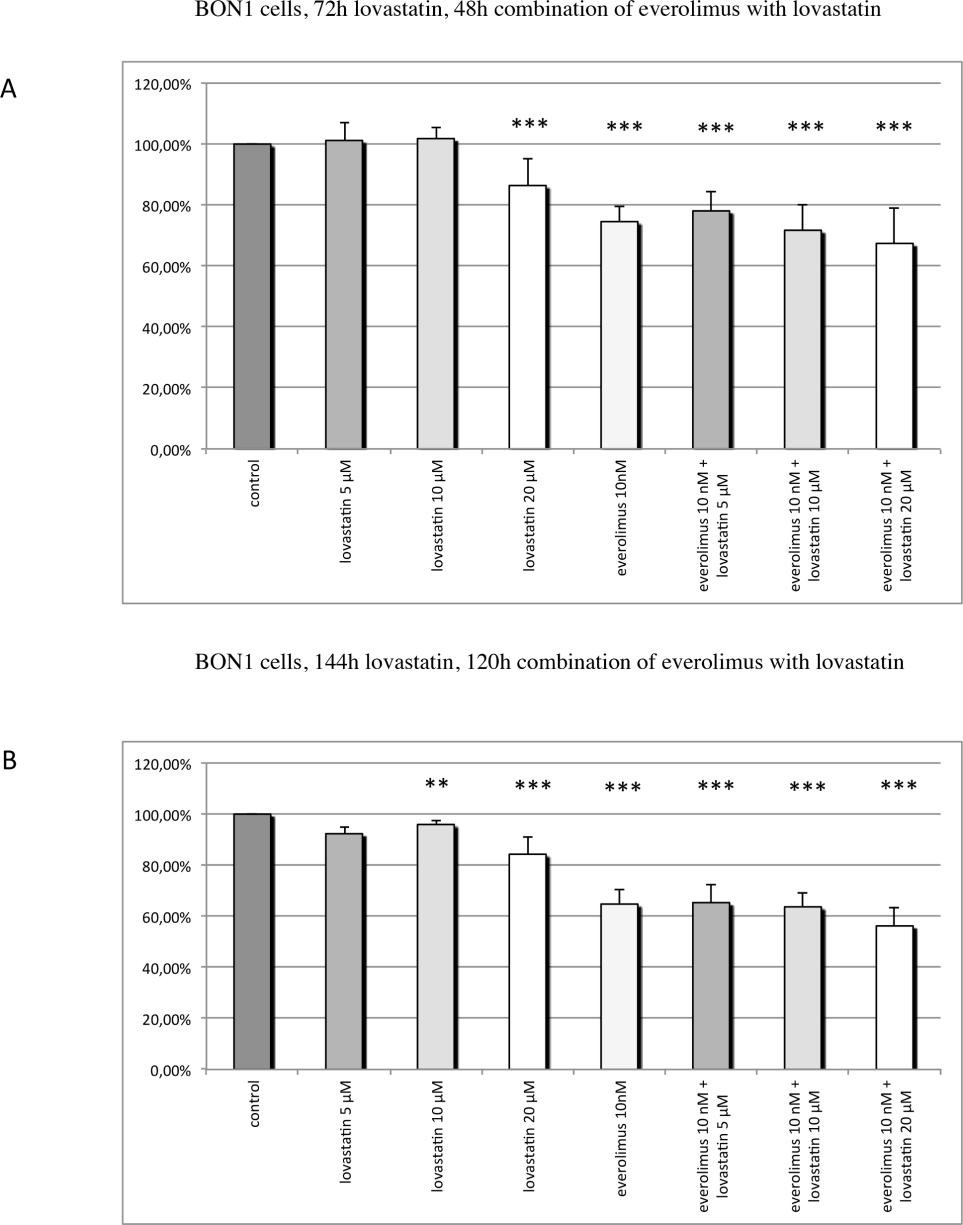
Cell viability in BON1 cells: BON1 cells were pre-treated with lovastatin for 24h before everolimus was added, and the combination of both drugs was incubated for 48h (A) or 120h (B). The single drugs were incubated for the same time as in combination. The mean percentage of cell viability, relative to the untreated control, ± SEM (error bars) is shown. (A) Treatment with 5–10 μM lovastatin had no significant effect on BON1 cell viability. Treatment with 20 μM lovastatin or with 10 nM everolimus alone significantly decreased BON1 cell viability. There was no additive effect of both drugs. (B) Treatment with 5–10 μM lovastatin had no significant effect on BON1 cell viability. Treatment with 20 μM lovastatin or with 10 nM everolimus separately significantly reduced BON1 cell viability; combination treatment showed no additive effect. * P ≤ 0.05, ** P ≤ 0.01, *** P ≤ 0.001 (compared to the control); @ P ≤ 0.05, @@ P ≤ 0.01, @@@ P ≤ 0.001 (combination compared to each drug separately).

**Fig 3:**
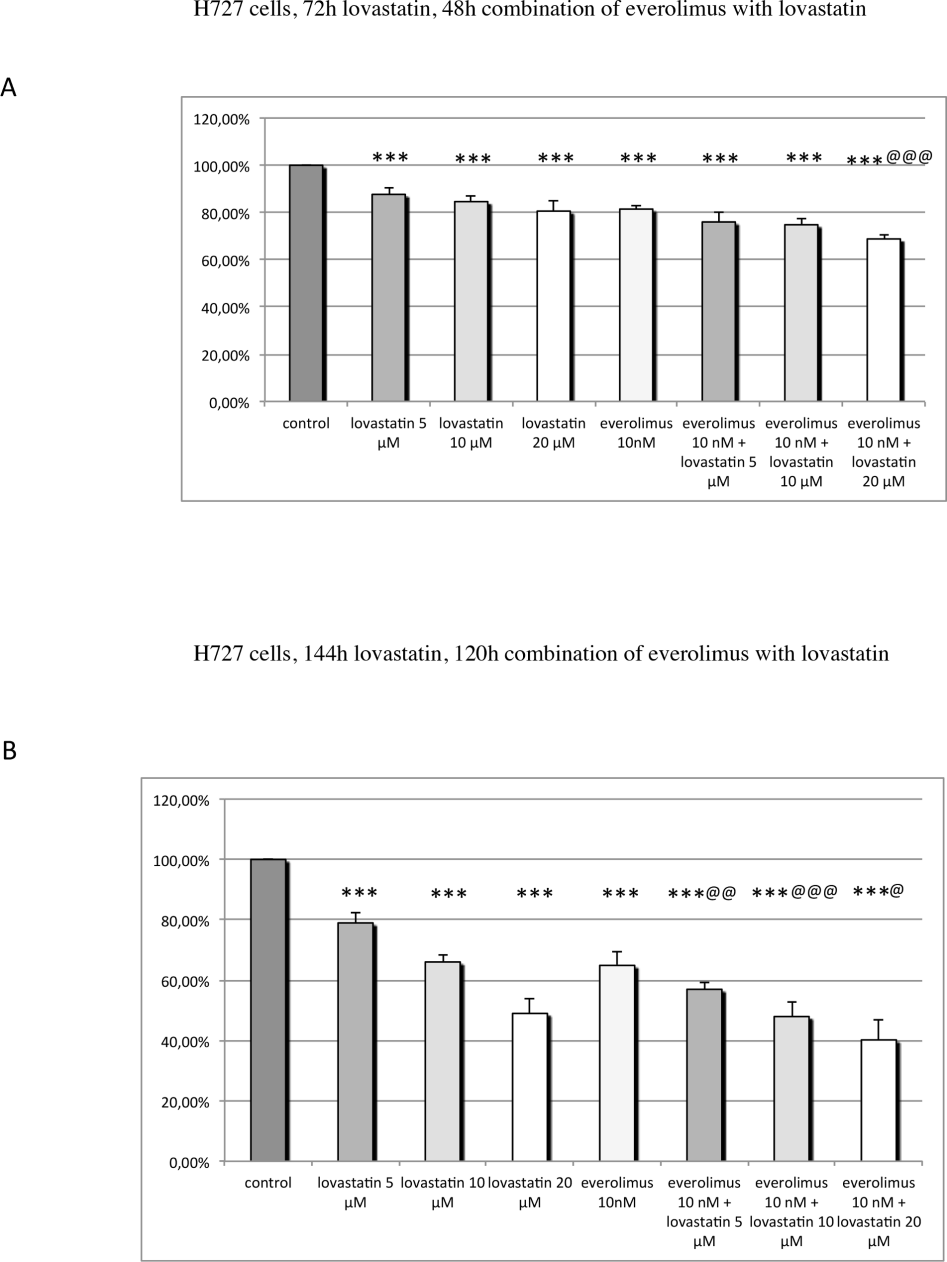
Cell viability in H727 cells: H727 cells were pre-treated with lovastatin for 24h before everolimus was added, and the combination of both drugs was incubated for 48h (A) or 120h (B). The single drugs were incubated for the same time as in combination. The mean percentage of cell viability, relative to the untreated control, ± SEM (error bars) is shown. (A) Treatment with 5–20 μM lovastatin or with 10 nM everolimus alone significantly decreased H727 cell viability. Combination treatment with 20 μM lovastatin and 10 nM everolimus significantly more strongly reduced cell viability than each drug separately. (B) Treatment with 5–20 μM lovastatin or with 10 nM everolimus separately significantly reduced H727 cell viability. The combination of 5–20 μM lovastatin with 10 nM everolimus significantly more strongly reduced cell viability compared to each drug given separately. * P ≤ 0.05, ** P ≤ 0.01, *** P ≤ 0.001 (compared to the control); @ P ≤ 0.05, @@ P ≤ 0.01, @@@ P ≤ 0.001 (combination compared to each drug separately).

**Fig 4:**
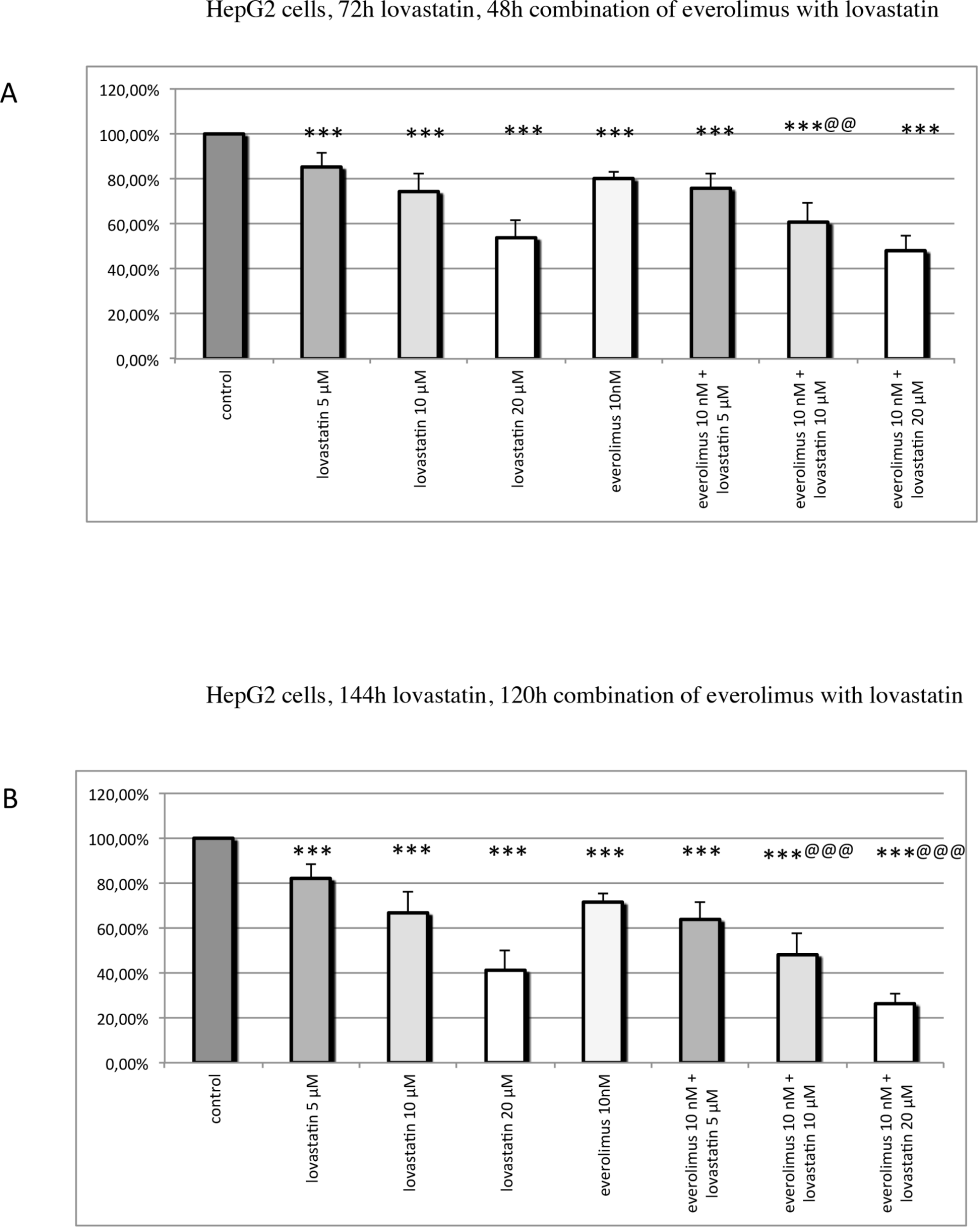
Cell viability in HepG2 cells: HepG2 cells were pre-treated with lovastatin for 24h before everolimus was added, and the combination of both drugs was incubated for 48h (A) or 120h (B). The single drugs were incubated for the same time as in combination. The mean percentage of cell viability, relative to the untreated control, ± SEM (error bars) is shown. (A) Treatment with 5–20 μM lovastatin or with 10 nM everolimus alone significantly decreased HepG2 cell viability. Combination treatment with 10 μM lovastatin and 10 nM everolimus significantly more strongly reduced cell viability than each drug separately. (B) Treatment with 5–20 μM lovastatin or with 10 nM everolimus separately significantly decreased HepG2 cell viability. Combination treatment with 10–20 μM lovastatin and 10 nM everolimus significantly more strongly reduced cell viability compared to each drug given alone. * P ≤ 0.05, ** P ≤ 0.01, *** P ≤ 0.001 (compared to the control); @ P ≤ 0.05, @@ P ≤ 0.01, @@@ P ≤ 0.001 (combination compared to each drug separately).

**Fig 5:**
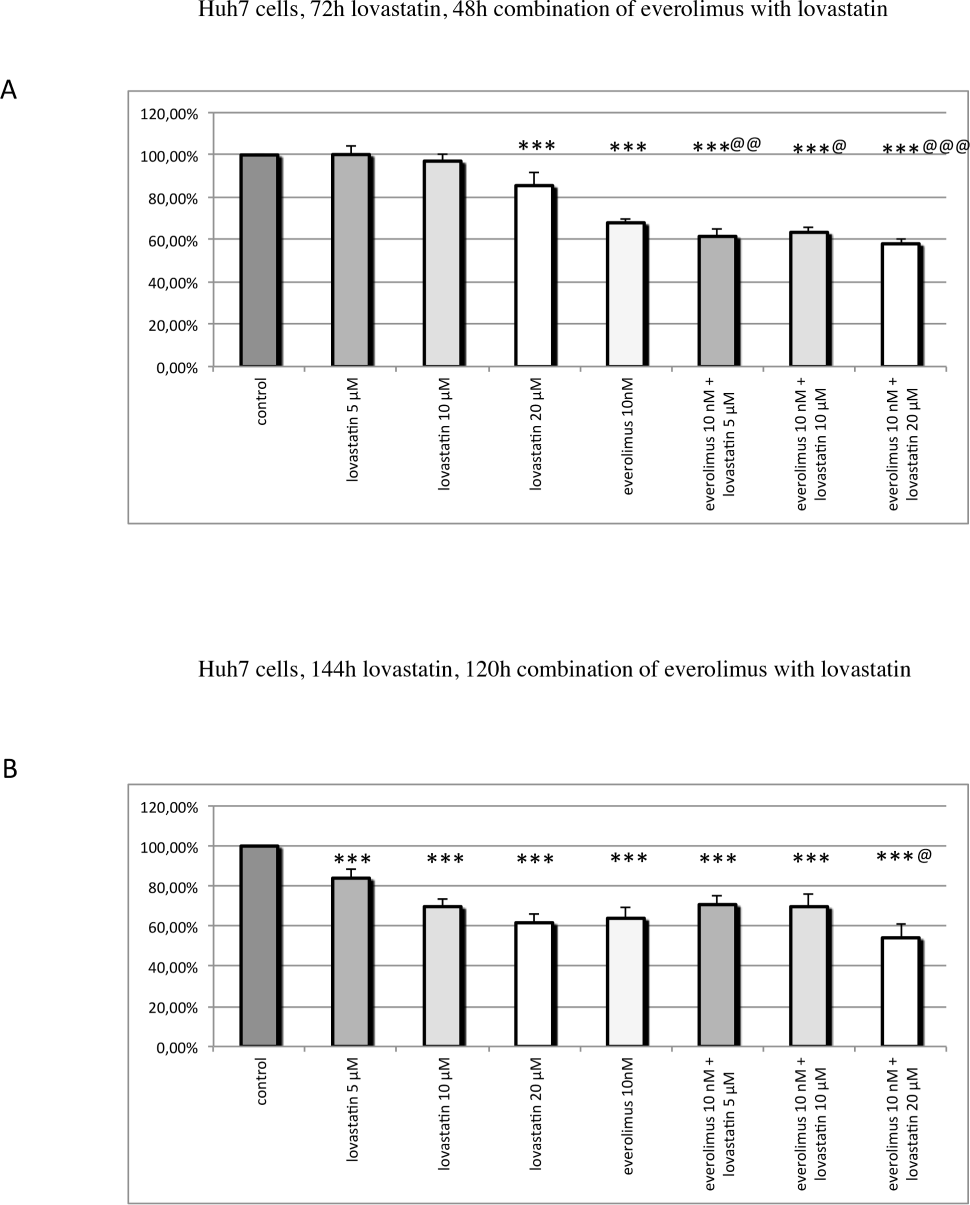
Cell viability in Huh7 cells: Huh7 cells were pre-treated with lovastatin for 24h before everolimus was added, and the combination of both drugs was incubated for 48h (A) or 120h (B). The single drugs were incubated for the same time as in combination. The mean percentage of cell viability, relative to the untreated control, ± SEM (error bars) is shown. (A) Treatment with 20 μM lovastatin or with 10 nM everolimus separately significantly reduced Huh7 cell viability. Combination treatment with 5–20 μM lovastatin and 10 nM everolimus significantly added to the effect of each drug alone. (B) Treatment with 5–20 μM lovastatin or with 10 nM everolimus alone significantly decreased Huh7 cell viability. Combination treatment with 20 μM lovastatin and 10 nM everolimus was significantly more effective than each drug separately. * P ≤ 0.05, ** P ≤ 0.01, *** P ≤ 0.001 (compared to the control); @ P ≤ 0.05, @@ P ≤ 0.01, @@@ P ≤ 0.001 (combination compared to each drug separately).

**Fig 6:**
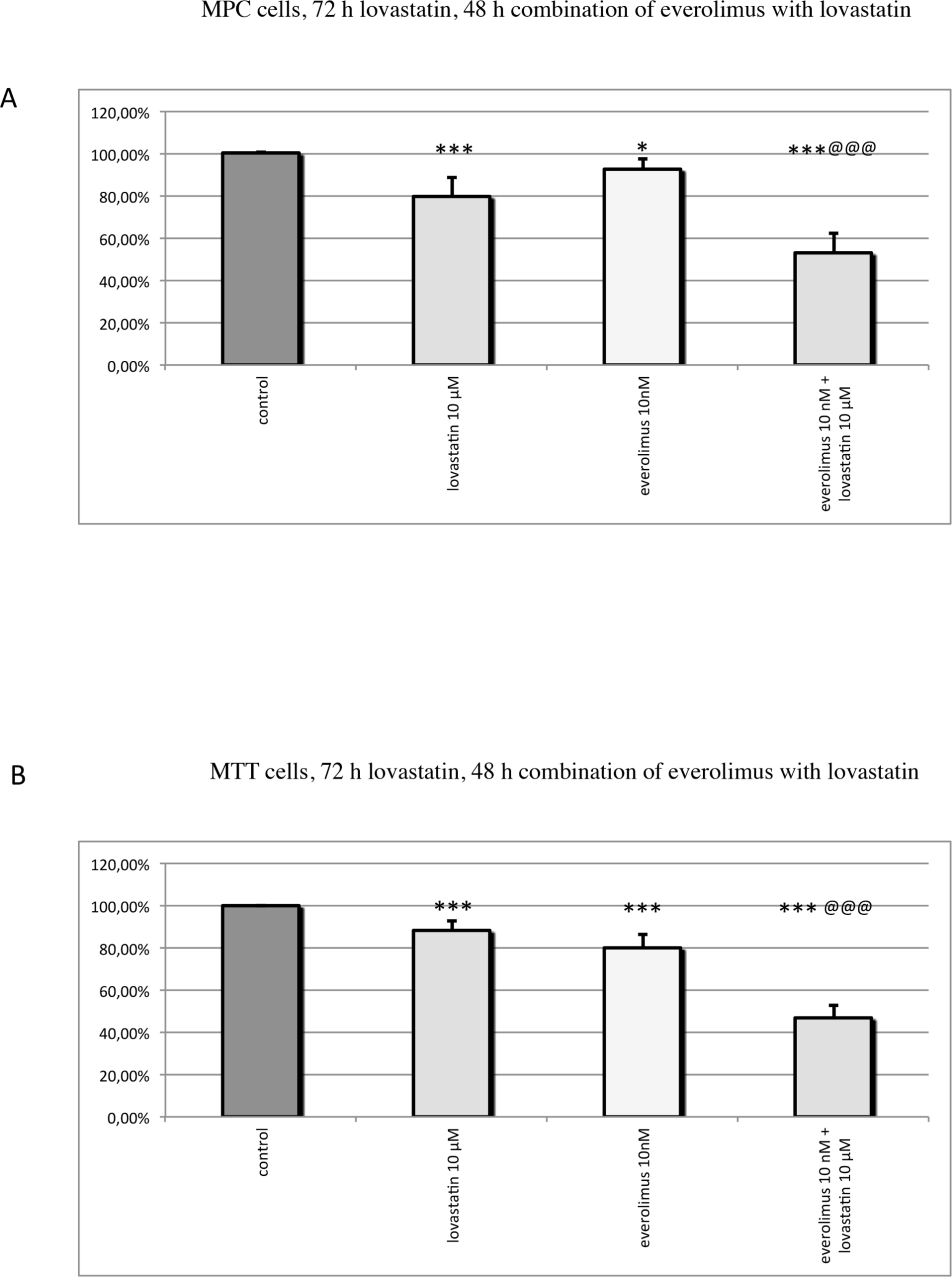
Cell viability in MPC (A) and MTT (B) cells: MPC and MTT cells were pre-treated with lovastatin for 24h before everolimus was added, and the combination of both drugs was incubated for 48h. The single drugs were incubated for the same time as in combination. The mean percentage of cell viability, relative to the untreated control, ± SEM (error bars) is shown. Treatment with 10 μM lovastatin or with 10 nM everolimus separately significantly reduced MPC and MTT cell viability, compared to the control. 48h combination treatment with 10 μM lovastatin and 10 nM everolimus significantly more potently decreased MPC and MTT cell viability compared to each drug given separately. * P ≤ 0.05, ** P ≤ 0.01, *** P ≤ 0.001 (compared to the control); @ P ≤ 0.05, @@ P ≤ 0.01, @@@ P ≤ 0.001 (combination compared to each drug separately).

BON1, GOT, H727, HepG2, Huh7, MPC and MTT cells were treated either with lovastatin or everolimus on their own or with the combination of both drugs in different doses for various incubation periods. The *combination-treated cells were always pre-treated with lovastatin for 24h and afterwards treated with the combination of both drugs for 48h or 120h*. For comparability reasons, the single drugs were incubated for the same time as in combination. Cell viability was measured. The mean percentage of cell viability, relative to the untreated control, ± SEM, after treatment with lovastatin, everolimus, or the combination, are shown in Figs 2, 3, 4, 5 and 6.

### BON1 cells

At both incubation periods, treatment with 20 μM lovastatin or with 10 nM everolimus alone significantly decreased BON1 cell viability. (144h treatment with 10 μM lovastatin very slightly, but statistically significantly, decreased cell viability.) Lower concentrations of lovastatin had no significant effects on cell viability. There was no additive effect of 5–20 μM lovastatin in combination with 10 nM everolimus (Fig 2).

### GOT cells

At both incubation times, there was no significant effect of 5–10 μM lovastatin on GOT cell viability, while treatment with 20 μM lovastatin for 144h significantly decreased cell viability; 48h or 120h treatment with 10 nM everolimus alone significantly decreased cell viability: 48h or 120h combination treatment did not significantly add to the effect of everolimus alone (data not shown).

### H727 cells

At both incubation periods, treatment with 5–20 μM lovastatin or with 10 nM everolimus alone significantly dose- and time-dependently decreased H727 cell viability (Fig 3); the combination of 20 μM lovastatin with 10 nM everolimus for 48h significantly more strongly decreased cell viability, compared to each drug given separately (Fig 3A): 120h of combination treatment with 5–20 μM lovastatin and 10 nM everolimus significantly more strongly reduced cell viability, compared to each drug separately (Fig 3B).

### HepG2 cells

At both incubation times, treatment with 5–20 μM lovastatin or with 10 nM everolimus separately significantly decreased HepG2 cell viability (Fig 4). 48h combination treatment with 10 μM lovastatin and 10 nM everolimus showed an additive effect with significantly stronger reduction of cell viability compared to each drug separately (Fig 4A): 120h combination of 10–20 μM lovastatin with 10 nM everolimus also showed an additive effect on cell viability (Fig 4B).

### Huh7 cells

72h treatment with 20 μM lovastatin or 144h treatment with 5–20 μM lovastatin significantly reduced Huh7 cell viability; at both incubation periods, treatment with 10 nM everolimus alone significantly decreased Huh7 cell viability (Fig 5). The combination treatment with 5–20 μM lovastatin and 10 nM everolimus for 48h was slightly, but statistically significantly, more effective than each drug separately (Fig 5A): 120h of combination treatment with 20 μM lovastatin and 10 nM everolimus slightly, but statistically significantly, more strongly reduced cell viability than each drug separately (Fig 5B).

### MPC and MTT cells

In both mouse pheochromocytoma cell lines MPC and MTT, 72h single treatment with clinically relevant doses of lovastatin or everolimus significantly reduced cell viability, as previously published [24]; we now confirm that 72h treatment with 10 μM lovastatin and 48h treatment with 10 nM everolimus separately significantly reduced MPC and MTT cell viability, but additionally combination treatment with 10 μM lovastatin and 10 nM everolimus for 48h significantly more potently decreased MPC and MTT cell viability compared to each drug separately (Fig 6).

### Western blots: Signaling pathways (Fig 7)

To study the effects of lovastatin, everolimus or the combination on EGFR, ERK, AKT and mTORC1/p70S6K signaling, Western blots of BON1, H727, HepG2 and Huh7 cells were performed. According to the results above, BON1 cells behave similarly to GOT cells, and represent a cell line in which lovastatin alone has only minor inhibitory effects on cell viability at high doses, but everolimus is effective and the combination shows no additive effect. H727, HepG2 and Huh7 cells represent cells in which lovastatin *and* everolimus are effective, and the combination works additively. For combination treatment, cells were always pre-treated with 20 μM lovastatin for 24h before 10 nM everolimus was added, and the combination of both drugs was incubated for 48h. Table 1 shows the mean percentage of absolute pEGFR, pERK, pAKT, and pp70S6K expression, relative to the untreated control, +/- SEM in the different cell lines after different drug treatments.

**Fig 7:**
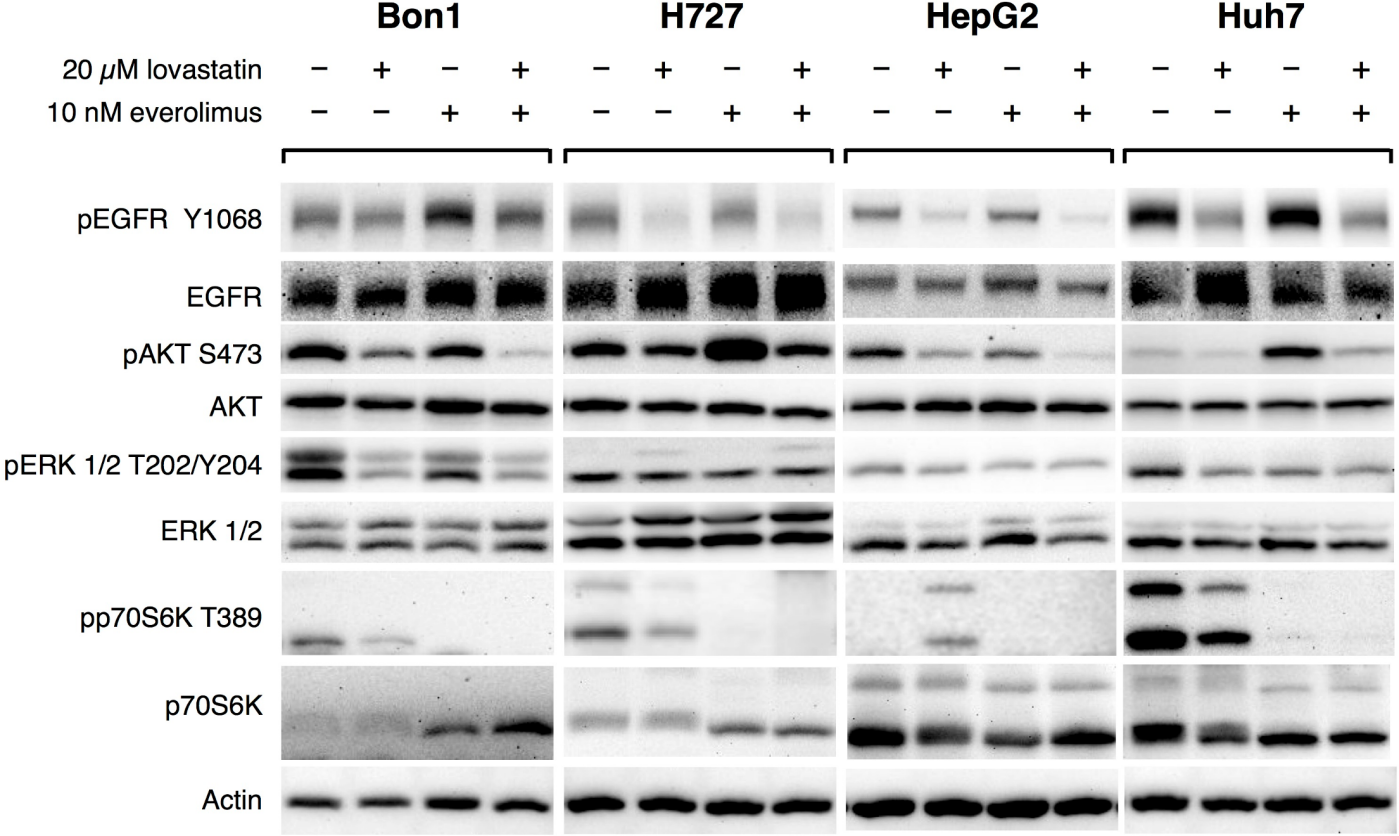
Representative Western blots: Effects of lovastatin and everolimus alone and in combination on EGFR, AKT, ERK and p70S6K signaling in BON1, H727, HepG2 and Huh7 cells: pEGFR was decreased in all cell lines after lovastatin treatment compared to the control—this was least in BON1 cells and increasingly stronger in Huh7, H727 and HepG2 cells. Apart from a clear up-regulation in BON1 cells after treatment with everolimus, everolimus hardly changed pEGFR in H727, HepG2 or Huh7 cells, compared to the control. Except for a very slight increase of pEGFR in BON1 cells, combination treatment decreased pEGFR in H727, HepG2 and Huh7 cells, compared to the control. pAKT was consistently suppressed in all cell lines after lovastatin treatment. After treatment with everolimus, there was a decrease of pAKT in BON1 and HepG2 cells, but a strong increase in H727 and Huh7 cells. Combination treatment led to a stronger suppression of pAKT than everolimus alone in all cell lines, and reduced pAKT compared to the control in BON1, H727 and HepG2. In Huh7 cells, combination treatment clearly attenuated the everolimus-induced increase of pAKT. Lovastatin treatment was accompanied by a strong decrease of pERK in BON1 cells, a clearly milder decrease in HepG2 and Huh7 cells, and no change of pERK in H727 cells. Everolimus led to a mild to moderate decrease of pERK in all cell lines. Apart from a slight increase of pERK in H727 cells, combination treatment was associated with suppression of pERK in BON1, HepG2 and Huh7 cells. Lovastatin treatment was accompanied by a very strong increase of pp70S6K in HepG2 cells. In BON1, H727 and Huh7 cells lovastatin led to a mild to moderate decrease of pp70S6K. Everolimus and combination treatment suppressed pp70S6K in all cell lines much more strongly than lovastatin did. Combination treatment completely inhibited p70S6K signaling in BON1, H727 and Huh7 cells, and clearly attenuated the lovastatin-induced pp70S6K up-regulation in HepG2 cells.

**Table 1:** Signaling pathways in BON1, H727, HepG2 and Huh7 cells. Effects of 48h combination treatment with 20 μM lovastatin and 10 nM everolimus after 24h pre-treatment with lovastatin on signaling pathways in BON1, H727, HepG2 and Huh7 cells compared to treatment with each drug separately. Data are shown as mean percentage of the absolute phospho-protein expression, relative to the untreated control, ± SEM.

pEGFR	20 μM lovastatin for 72h	10 nM everolimus for 48h	72h 20 μM lovastatin plus 48h 10 nM everolimus
BON1	77% ± 11%	153% ± 14%	110% ± 25%
H727	55% ± 9%	107% ± 12%	49% ± 10%
HepG2	54% ± 18%	81% ± 20%	53% ± 21%
Huh7	65% ± 13%	93% ± 10%	64% ± 14%
**pAKT**			
BON1	43% ± 6%	74% ± 17%	28% ± 12%
H727	57% ± 16%	190% ± 76%	58% ± 19%
HepG2	55% ± 30%	39% ± 9%	16% ± 4%
Huh7	75% ± 25%	221% ± 25%	152% ± 49%
**pERK**			
BON1	40% ± 11%	88% ± 19%	41% ± 13%
H727	94% ± 15%	85% ± 19%	127% ± 27%
HepG2	60% ± 19%	63% ± 19%	50% ± 14%
Huh7	56% ± 6%	64% ± 6%	62% ± 3%
**pp70S6K**			
BON1	80% ± 11%	16% ± 8%	4% ± 2%
H727	59% ± 17%	22% ± 9%	9% ± 3%
HepG2	598% ± 25%	81% ± 19%	129% ± 23%
Huh7	77% ± 7%	8% ± 4%	1% ± 1%

Representative Western blots for each cell line and signaling pathway are shown in Fig 7.

### EGFR signaling / pEGFR

In all cell lines investigated treatment with lovastatin decreased EGFR activity (pEGFR): in BON1 cells pEGFR was least decreased; in H727, HepG2 and Huh7 cells, there was a clear suppression of pEGFR after treatment with lovastatin.

In BON1 cells, pEGFR was increased after treatment with everolimus alone. In H727, HepG2 and Huh7 cells, there was no relevant alteration of EGFR signaling after treatment with everolimus, compared to the control.

In BON1 cells combination treatment slightly increased pEGFR. In H727, HepG2 and Huh7 cells combination treatment decreased pEGFR to a similar extent as lovastatin treatment, compared to the control (Table 1).

### EGFR downstream signaling cascades ERK, AKT, and mTORC1/p70S6K signaling

ERK: In BON1, HepG2 and Huh7 cells, activated ERK (pERK) was mildly to moderately down-regulated after treatment with lovastatin, compared to the control. In H727 cells, no relevant alteration of pERK was found after treatment with lovastatin.

In all cell lines investigated, pERK was slightly decreased after treatment with everolimus alone.

In BON1, HepG2 and Huh7 cells, but not in H727 cells, combination treatment was associated with a mild to moderate decrease of pERK, compared to the control (Table 1).

AKT: In all cell lines investigated, there was found a mild to moderate inhibition of AKT signaling (pAKT) after treatment with lovastatin alone.

In BON1 and HepG2 cells, single treatment with everolimus mildly to moderately decreased pAKT. In H727 and Huh7 cells, pAKT was strongly increased after single treatment with everolimus.

In BON1, H727 and HepG2 cells, combination treatment was accompanied by a moderate to strong decrease of pAKT–by a stronger decrease, compared to treatment with everolimus separately. In Huh7 cells, the everolimus-induced up-regulation of pAKT was not completely abrogated, but attenuated, after combination treatment (Table 1).

MTORC1 / p70S6K T389: In BON1, H727 and Huh7 cells, the mTORC1 target pp70S6K was mildly to moderately reduced after treatment with lovastatin. In HepG2 cells, pp70S6K was very strongly increased after treatment with lovastatin. In all cell lines investigated, treatment with everolimus inhibited p70S6K signaling—to a much stronger extent than lovastatin treatment.

In BON1, H727 and Huh7 cells, combination treatment completely inhibited p70S6K signaling. In HepG2 cells, which showed a low basic activity of p70S6K signaling, combination treatment did not completely abolish, but clearly attenuated lovastatin-induced up-regulation of pp70S6K (Table 1).

**Fig 8:**
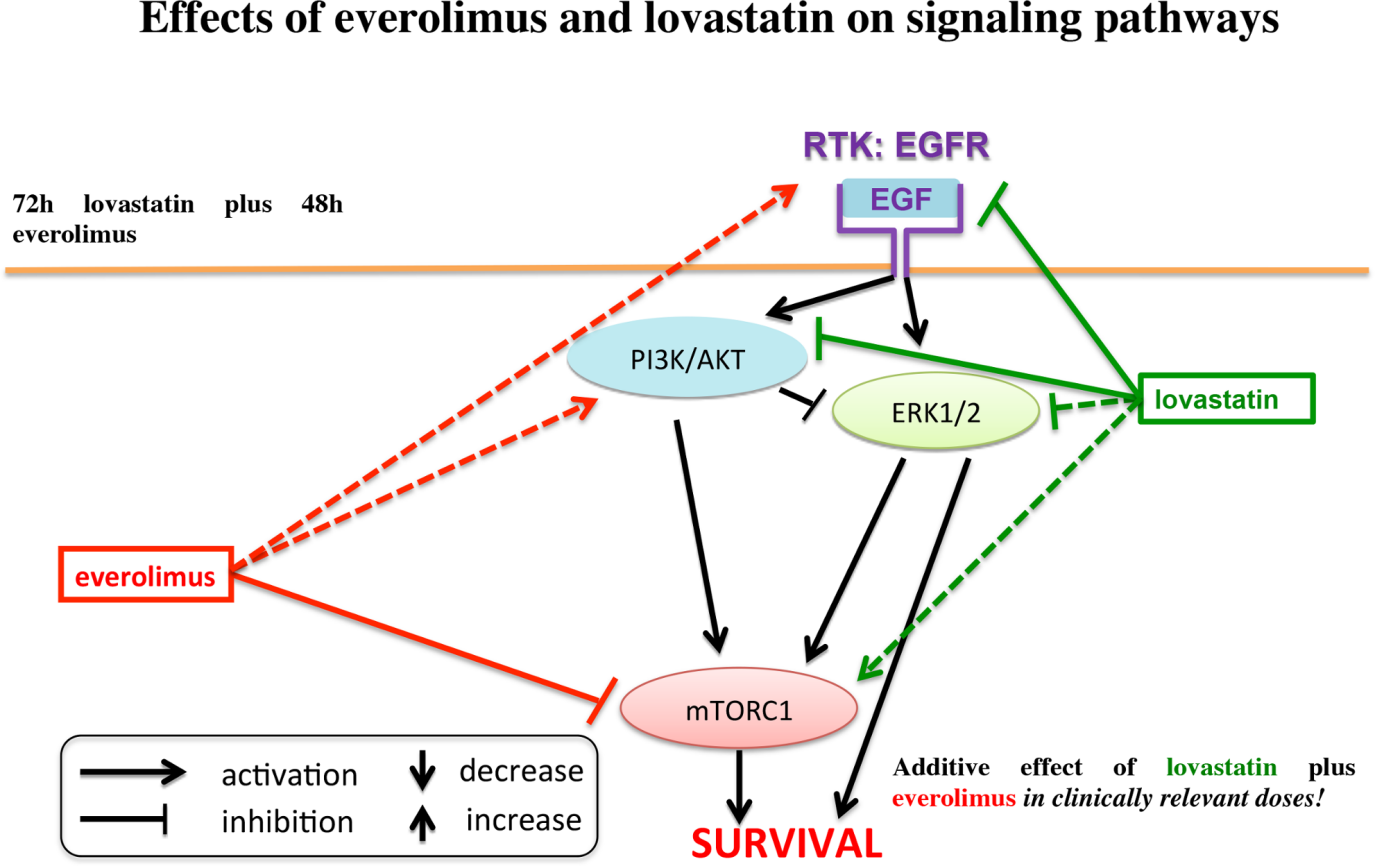
The effects of lovastatin and everolimus on the signaling pathways found in the present study: The dashed arrows show inconsistent effects on the signaling pathways in different cell lines. In all cell lines investigated, lovastatin led to suppression of EGFR and AKT signaling (green lines). Moreover, lovastatin was associated with ERK inhibition in BON1, HepG2 and Huh7 cell lines, but not in H727 cells (no effect on ERK signaling) (dashed green line). While leading to a massive increase of pp70S6K in HepG2 cells, lovastatin was accompanied by a mild to moderate decrease of pp70S6K in BON1, H727 and Huh7 cells (green dashed arrow). Everolimus, in contrast, always led to mTORC1/p70S6K inhibition (red line). However, everolimus was inconsistently associated with an increase of EGFR and AKT signaling: everolimus was accompanied by a strong increase of pEGFR in BON1 cells. In H727, HepG2 and Huh7 cells EGFR signaling was not markedly affected by everolimus (red dashed arrow). In H727 and Huh7 cells, everolimus led to a strong increase of pAKT, but in BON1 and HepG2 cells to a decrease of pAKT (red dashed arrows).

The effects of lovastatin and everolimus on the signaling pathways found in the present study are summarized in Fig 8.

## Discussion

We have investigated the effects of lovastatin and everolimus separately and in combination on cell viability and signaling pathways of human pancreatic (BON1), midgut (GOT) and pulmonary (H727) NET, hepatic carcinoma (HepG2 and Huh7) and mouse pheochromocytoma (MPC and MTT) cells.[27]

### Why lovastatin was used

In our prior studies, we have already achieved positive results with lovastatin inhibiting pheochromocytoma cell viability *in vitro* and *in vivo* [24, 26].

### And why lovastatin specifically?

For pheochromocytoma cells, three different lipophilic and hydrophilic statins have already been tested: the three lipophilic statins lovastatin, simvastatin, and fluvastatin showed the highest inhibitory effects on cell viability and similar effects on MAPK signaling, apoptosis-associated proteins and spontaneous cell migration. However, direct comparison of the three most effective statins revealed a higher potency of simvastatin and fluvastatin compared to lovastatin [25]. Nevertheless, we continued to use lovastatin—as a lipophilic representative with anti-tumor potential—in order to compare the current data to our previous cell line studies.

### Lovastatin dose relevance

Blood levels up to 12.5 μM lovastatin are possible in humans and well-tolerated without dose-limiting toxicity, although doses >10 μM are difficult to achieve [27]. Doses between 5 μM and 10 μM are more likely to be achieved and are clinically relevant [27], such that these doses were used for our cell viability experiments. Moreover, we found a significant decrease of MPC and MTT cell viability at doses of 10 μM lovastatin after 72h, and in our MTT cell allograft mouse model, a *daily human standard dose of 80 mg lovastatin* (converted to an equivalent mouse dose) was found to significantly reduce tumor growth over time [26]. This suggests the transferability of these *in vitro* findings to *in vivo* experiments with standard dosing. For Western blot experiments, however, higher lovastatin doses were used to show the effects more clearly since the higher the amount of affected phospho-proteins the better the Western blot signal.

### Hypotheses

The primary hypothesis of significant reduction of cell viability by each drug separately was confirmed in H727, MPC, MTT, HepG2 and Huh7 cells; in contrast, it had to be rejected for BON1 and GOT cells. In the latter cells low-dose everolimus did indeed significantly reduce BON1 and GOT cell viability, but very high doses of lovastatin were necessary to significantly reduce BON1 and GOT cell viability.

The secondary hypothesis of an additive effect of both drugs at clinically relevant doses also applied to H727, MPC, MTT, HepG2 and Huh7 cells. The hypothesis was falsified for BON1 and GOT cells, being consistent with the absent effects observed for lovastatin separately in these cell lines.

The additive effect was dependent on the efficacy of each drug being consistent with the special feature of additive drug action: the sum of the separate drug effects is equal to the combined effect. Everolimus was similarly effective in all cell lines investigated. Therefore, the additive effect was primarily dependent on the effectiveness of lovastatin.

Very recently, a large clinical study has shown poor outcome in patients suffering from advanced hepatocellular carcinoma and treated with everolimus after failure of sorafenib [38]. Similarly, everolimus alone has been evaluated in a small number of patients with malignant pheochromocytoma/paraganglioma but there was no clinically significant tumor response [39].

In the face of these clinical data, our results suggest that *combinations* of targeted drugs, such as everolimus and lovastatin, may show additive inhibitory effects on cell viability which may be less obvious with the agents given individually. However, the variable reactivity of the different cell lines emphasizes the importance of analyzing the aberrant signaling pathways of each tumor to optimize molecular-targeted combination therapies. EGFR and its downstream signaling pathways ERK, AKT and mTORC1/p70S6K were investigated to understand the differences in drug efficacy depending on the cell line.

### EGFR signaling

In all cell lines investigated, treatment with lovastatin separately suppressed EGFR signaling (pEGFR). Subsequently, combination treatment more strongly suppressed pEGFR than everolimus alone or at least attenuated everolimus-induced EGFR activation. The degree of EGFR inhibition positively correlated with the efficacy of lovastatin and the additive effect in combination with everolimus: in BON1 cells, the mildest decrease of pEGFR was observed; stronger lovastatin-induced suppression of pEGFR and cell viability was found in H727, HepG2 and Huh7 cells. Lovastatin may inhibit EGFR and its downstream signaling cascades through inhibition of HMG-CoA reductase and depletion of mevalonate metabolites [40–42], required for EGFR function [42, 43]. Statins have previously been described to inhibit ligand-induced EGFR autophosphorylation, to enhance the activity of EGFR-inhibitor gefitinib in different cancer cell lines and primary tumor cultures, and improve progression free survival in NSCLC patients treated with EGFR-tyrosine-kinase-inhibitors [17–20, 22, 23]. Our results are concordant with a central role of EGFR inhibition in the mechanism of action of lovastatin.

### ERK and AKT and mTORC1/p70S6K

As described before for MPC and MTT cells [24, 25], pERK was decreased after single treatment with lovastatin in BON1, HepG2 and Huh7 cells. However, in H727 cells, lovastatin did not affect pERK, although it significantly decreased cell viability in these cells. Therefore, ERK inhibition may not consistently be responsible for the efficacy of lovastatin.

In all cell lines investigated, lovastatin treatment was accompanied by a suppression of pAKT. Accordingly, combination treatment more strongly suppressed pAKT than everolimus alone or at least attenuated everolimus-induced AKT activation, as similarly described before for the combination of gefitinib with lovastatin [17]. Consistent with previous studies [9–11], in H727 and Huh7 cells, there was an up-regulation of pAKT after everolimus treatment; in contrast, the everolimus-induced decrease of pAKT in BON1 and HepG2 cells found in the present study might be due to longer incubation times and be transient. A relevant effect on AKT signaling by lovastatin and simvastatin, respectively, has previously been shown for pheochromocytoma, head and neck squamous cell carcinoma, and T790M mutant NSCLC cell lines [17, 22, 24].

Everolimus constantly inhibited p70S6K signaling in all cell lines tested, and accordingly, combination treatment more strongly inhibited p70S6K signaling than lovastatin alone, or attenuated lovastatin-induced p70S6K activation.

In summary, combination treatment more strongly suppressed EGFR and AKT signaling than everolimus alone or at least attenuated everolimus-induced EGFR or AKT activation, and additionally, more strongly inhibited p70S6K signaling than lovastatin alone or attenuated lovastatin-induced p70S6K activation. Further studies will be necessary to confirm the postulated mechanisms of action by using specific inhibitors in short and long-term experiments.

Our overall conclusion is that (1) lovastatin and everolimus affect cell viability differently in different cancer cell lines; (2) that where effects were seen when the drug was used alone then an additive effect was seen with the combination; and (3) that the effects were broadly in agreement with the two agents affecting different signaling pathways. We suggest that these or similar combinations may be worth consideration in the treatment of various human neoplasms.

## Supporting Information

S1 Fig Original uncropped Western blot 05.12.14 pp70S6K Bon1, H727, HepG2, Huh7. (TIF)

S2 Fig Original uncropped Western blot 06.10.14 plus 27.10.14 pp70S6K Bon1 H727 HepG2 Huh7. (TIF)

S3 Fig Original uncropped Western blot 05.05.14 pp70S6K Huh7. (TIF)

S4 Fig Original uncropped Western blot 05.05.14 pp70S6K HepG2. (TIF)

S5 Fig Original uncropped Western blot 17.10.13 pp70S6K Huh7. (TIF)

S6 Fig Original uncropped Western Blot 17.10.13 pp70S6K HepG2. (TIF)

S7 Fig Original uncropped Western Blot 10.03.14 pp70S6K H727. (TIF)

S8 Fig Original uncropped Western Blot 28.10.13 pp70S6K H727. (TIF)

S9 Fig Original uncropped Western Blot 10.03.14 pp70S6K Bon1. (TIF)

S10 Fig Original uncropped Western Blot 02.10.13 plus 09.10.13 pp70S6K Bon1. (TIF)

S11 Fig Original uncropped Western Blot 26.09.13 pp70S6K H727 Huh7. (TIF)

S12 Fig Original uncropped Western Blot 17.10.13 pEGFR pAkt pErk Huh7. (TIF)

S13 Fig Original uncropped Western Blot 05.05.14 pEGFR pAkt pErk Huh7. (TIF)

S14 Fig Original uncropped Western Blot 05.05.14 pEGFR pAkt pErk HepG2. (TIF)

S15 Fig Original uncropped Western Blot 26.09.13 pEGFR pAkt pErk H727 Huh7. (TIF)

S16 Fig Original uncropped Western Blot 10.03.14 pEGFR pAkt pErk H727. (TIF)

S17 Fig Original uncropped Western Blot 02.10.13 plus 09.10.13 pEGFR pAkt pErk Bon1. (TIF)

S18 Fig Original uncropped Western Blot 10.03.14 pEGFR pAkt pErk Bon1. (TIF)

S19 Fig Original uncropped Western Blot 06.10.14 plus 27.10.14 pEGFR pAkt pErk Bon1 H727 HepG2 Huh7. (TIF)

S20 Fig Original uncropped Western Blot 05.12.14 pEGFR pAkt pErk Bon1 H727 HepG2 Huh7. (TIF)

S21 Fig Original uncropped Western Blot 17.10.13 pEGFR pAkt pErk HepG2. (TIF)

S22 Fig Original uncropped Western Blot 28.10.13 pEGFR pAkt pErk H727. (TIF)

S1 Table Cell viability raw data after the shorter drug-incubation time. Single values (at least 6 for each drug concentration per cell line experiment) are shown of the cell viability data after the shorter drug incubation time, normalized for comparison between experiments by dividing the raw data of each single experiment by the mean of the untreated simultaneous control.
(XLSX)

S2 Table Cell viability raw data after the longer drug-incubation time. Single values (at least 6 for each drug concentration per cell line experiment) are shown of the cell viability data after the longer drug incubation time, normalized for comparison between experiments by dividing the raw data of each single experiment by the mean of the untreated simultaneous control.
(XLSX)

S3 Table Western Blot data used for quantification. Single values of each Western blot for each protein and cell line line, normalized by dividing by the untreated control.
(XLSX)
