# Supplementary figures and images for: Additive Anti-Tumor Effects of Lovastatin and Everolimus In Vitro through Simultaneous Inhibition of Signaling Pathways
Source: PLoS One. 2015 Dec 4;10(12):e0143830. doi: 10.1371/journal.pone.0143830 (PMC4670204; doi:10.1371/journal.pone.0143830)

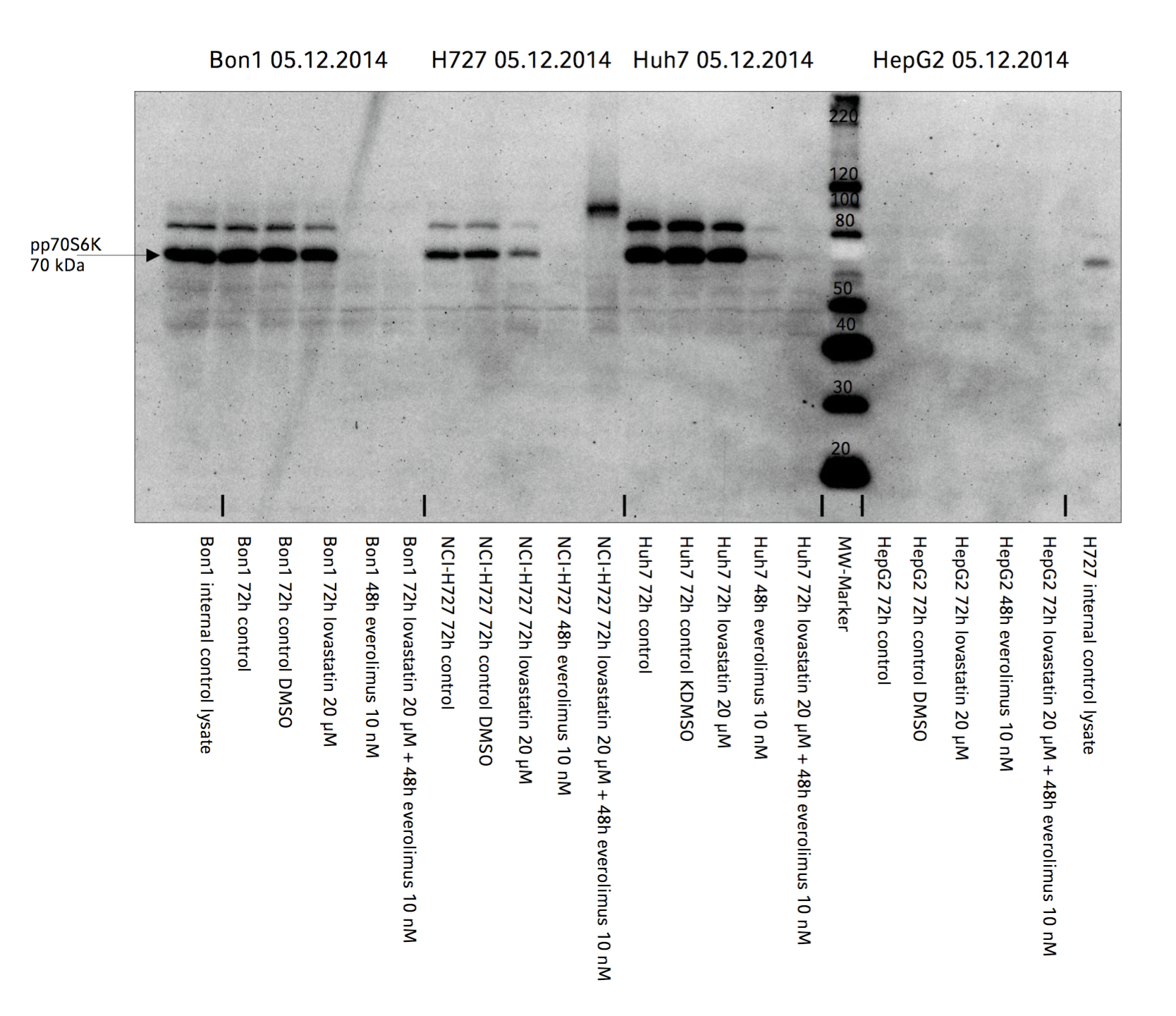

Supplement: S1 Fig — (TIF) [file pone.0143830.s001.tif]

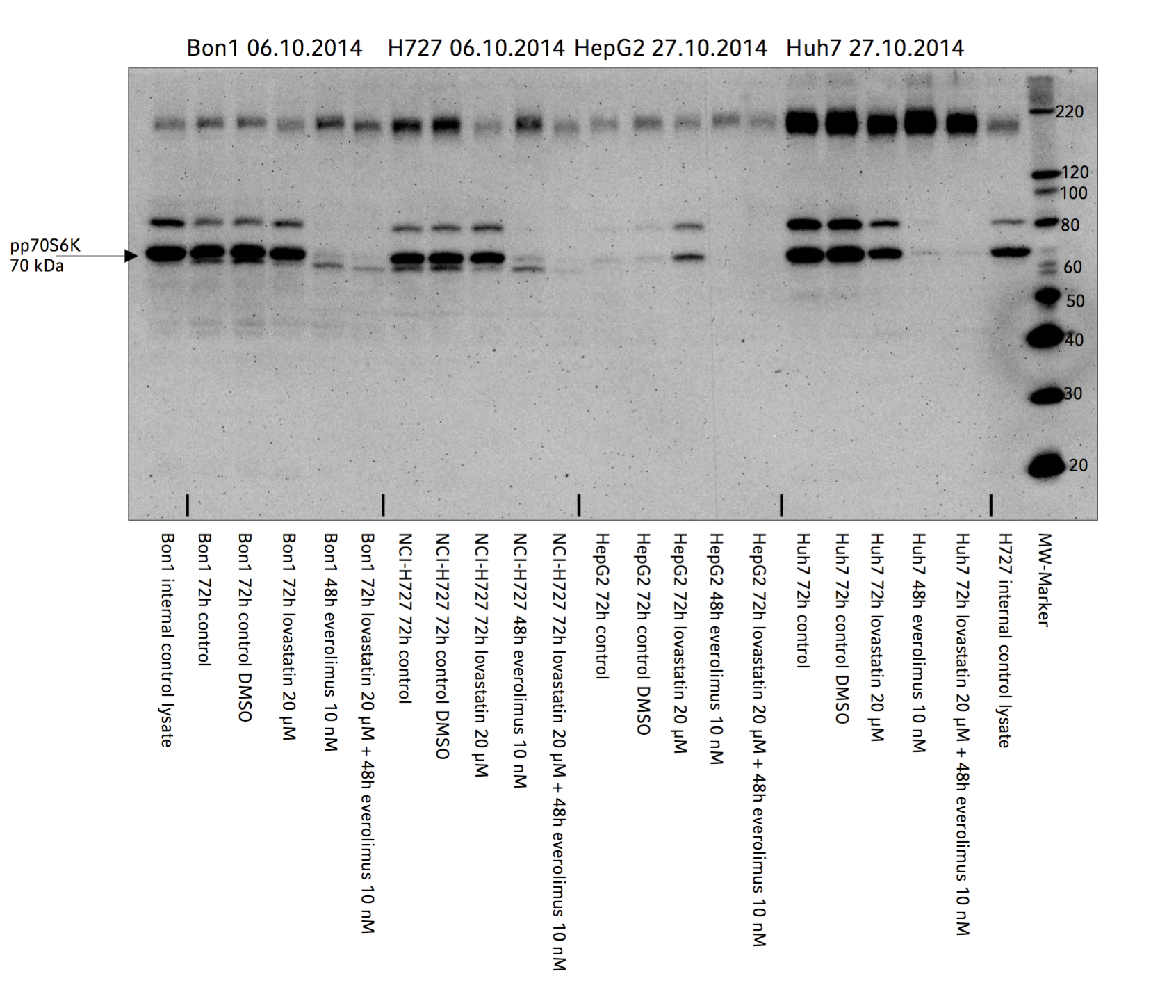

Supplement: S2 Fig — (TIF) [file pone.0143830.s002.tif]

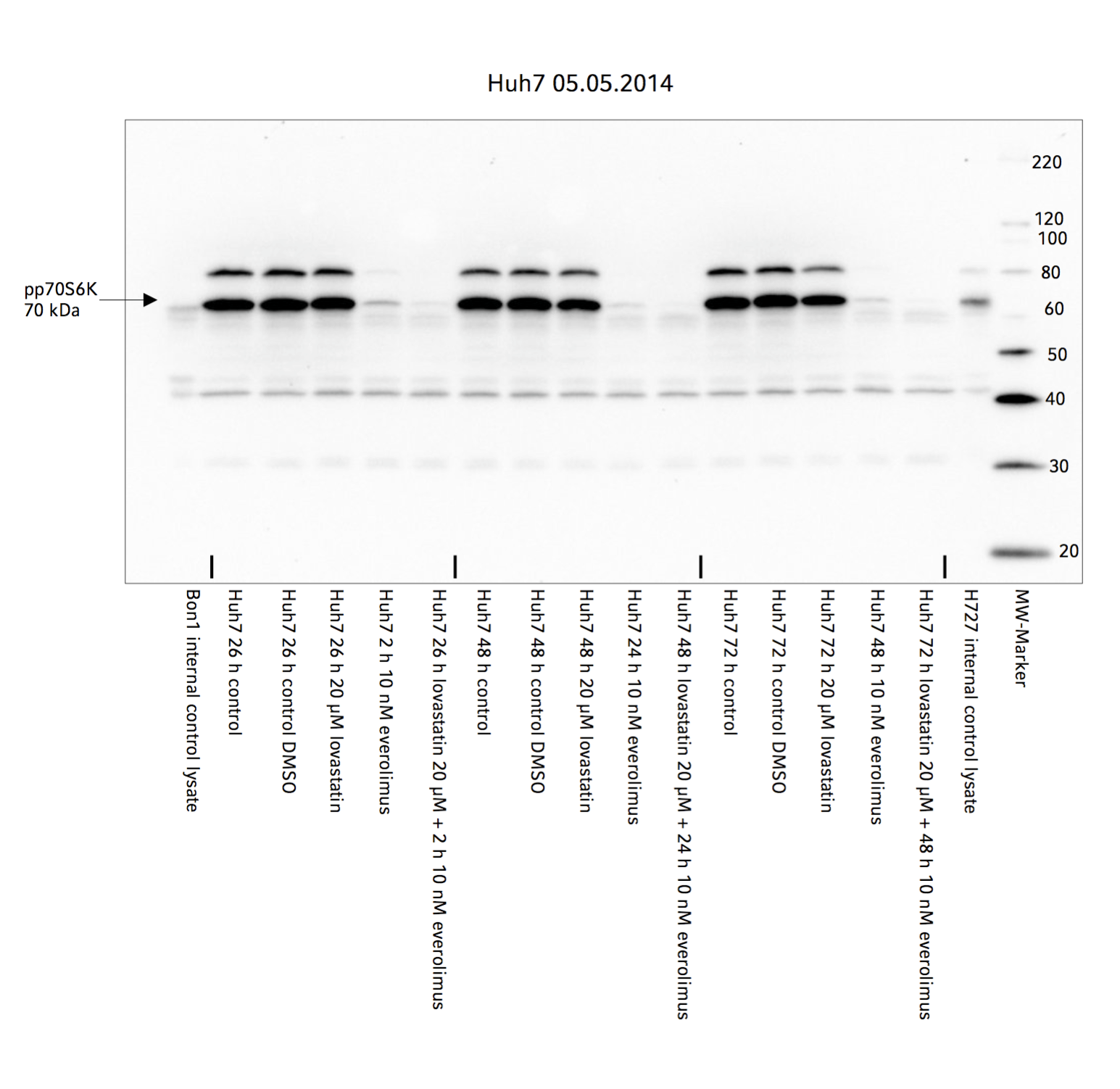

Supplement: S3 Fig — (TIF) [file pone.0143830.s003.tif]

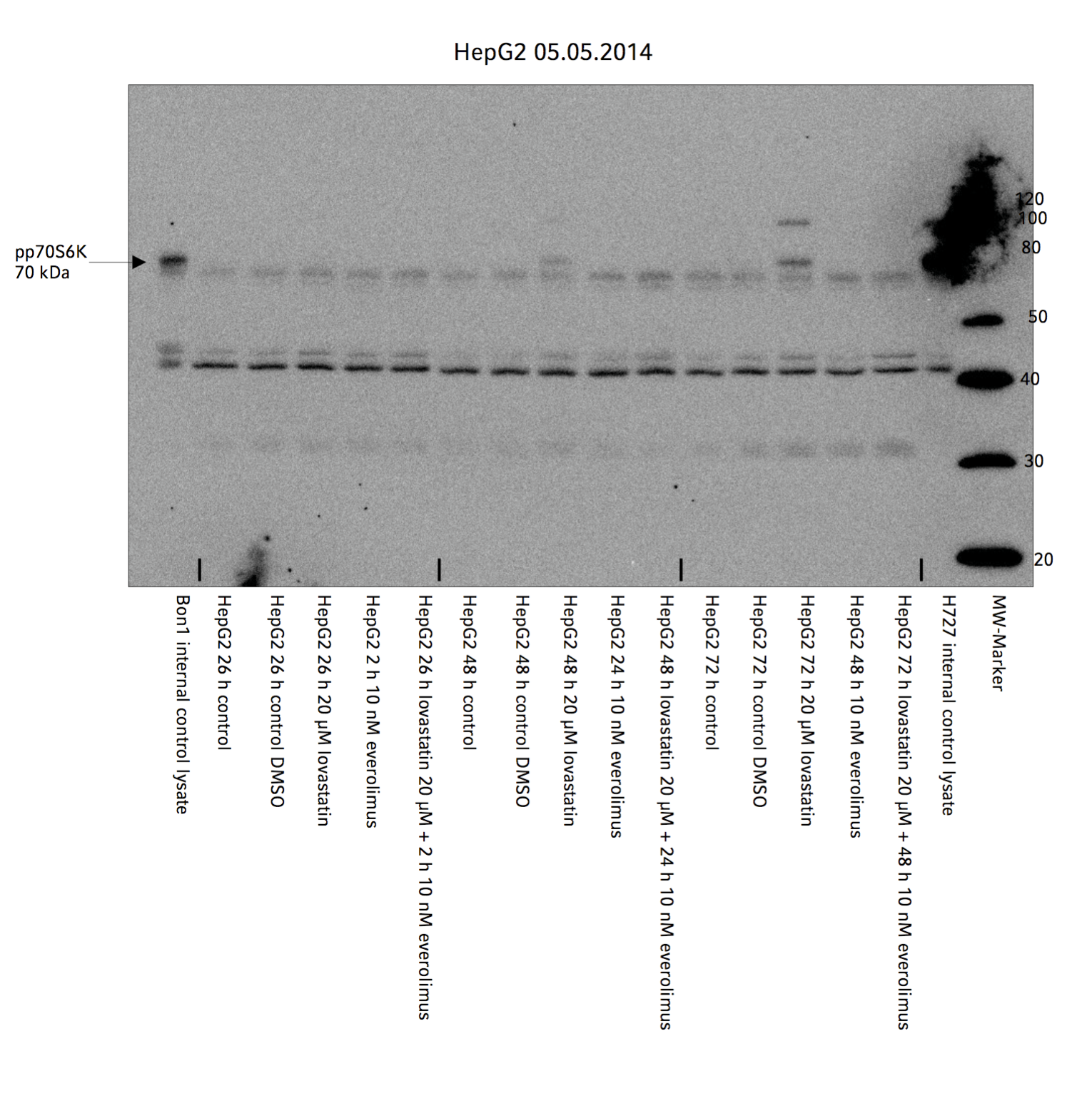

Supplement: S4 Fig — (TIF) [file pone.0143830.s004.tif]

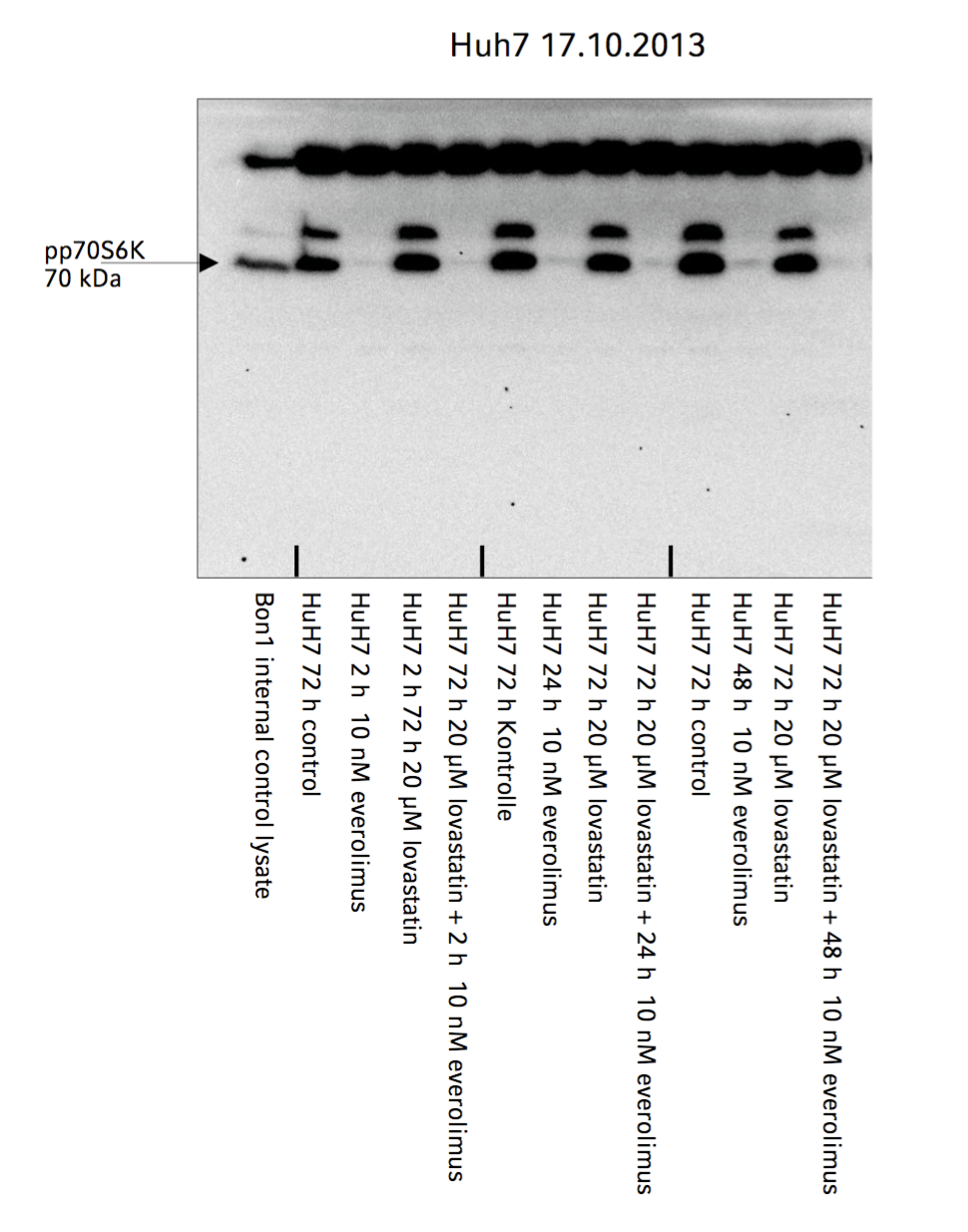

Supplement: S5 Fig — (TIF) [file pone.0143830.s005.tif]

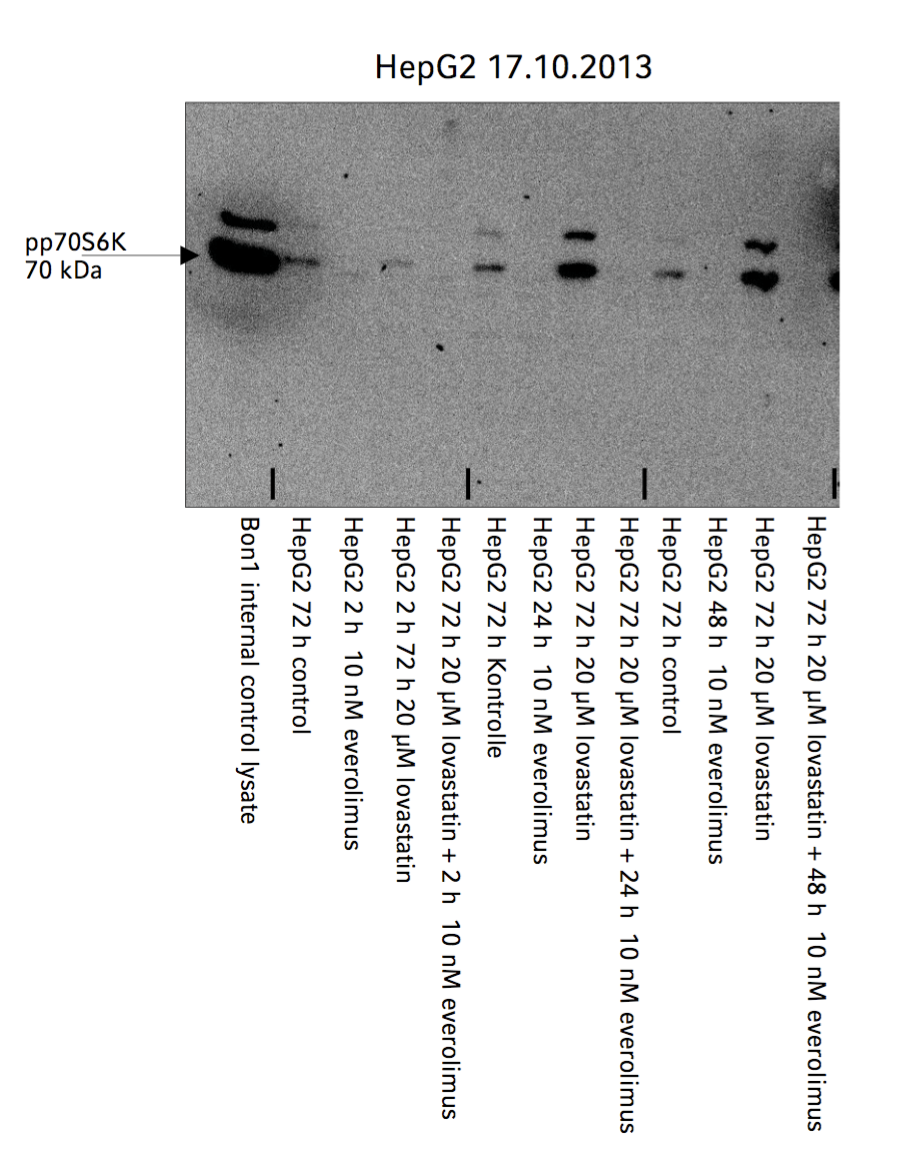

Supplement: S6 Fig — (TIF) [file pone.0143830.s006.tif]

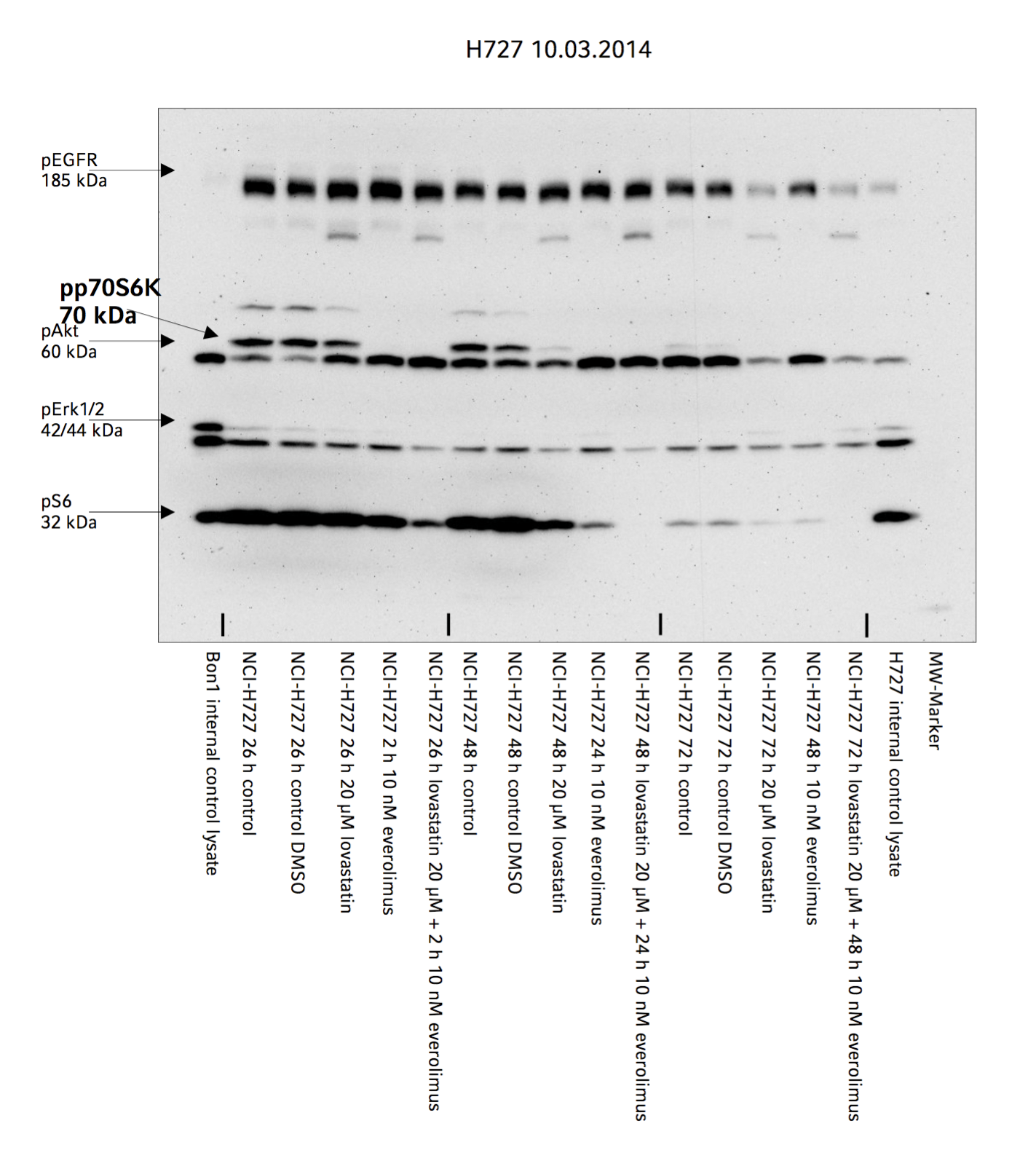

Supplement: S7 Fig — (TIF) [file pone.0143830.s007.tif]

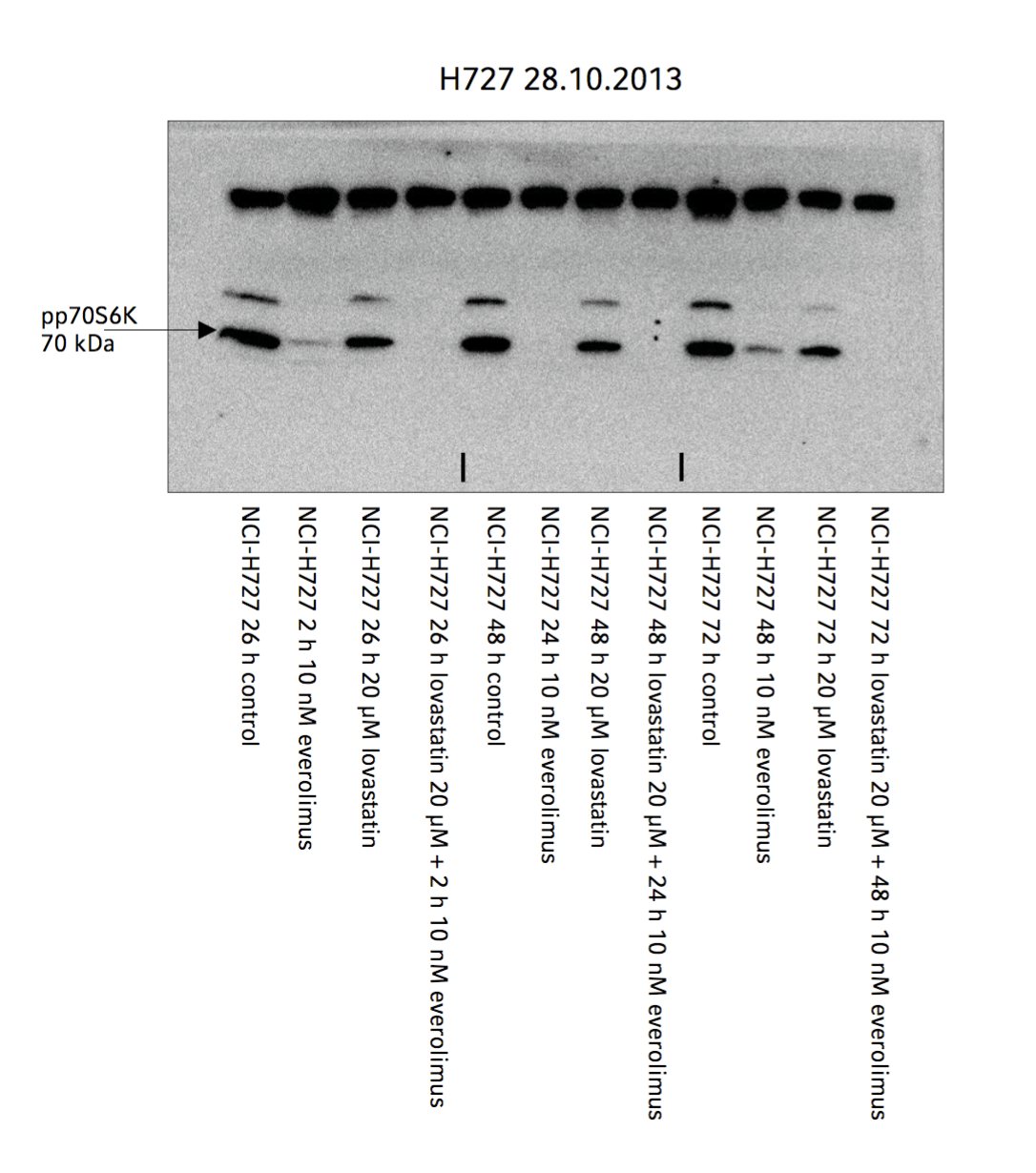

Supplement: S8 Fig — (TIF) [file pone.0143830.s008.tif]

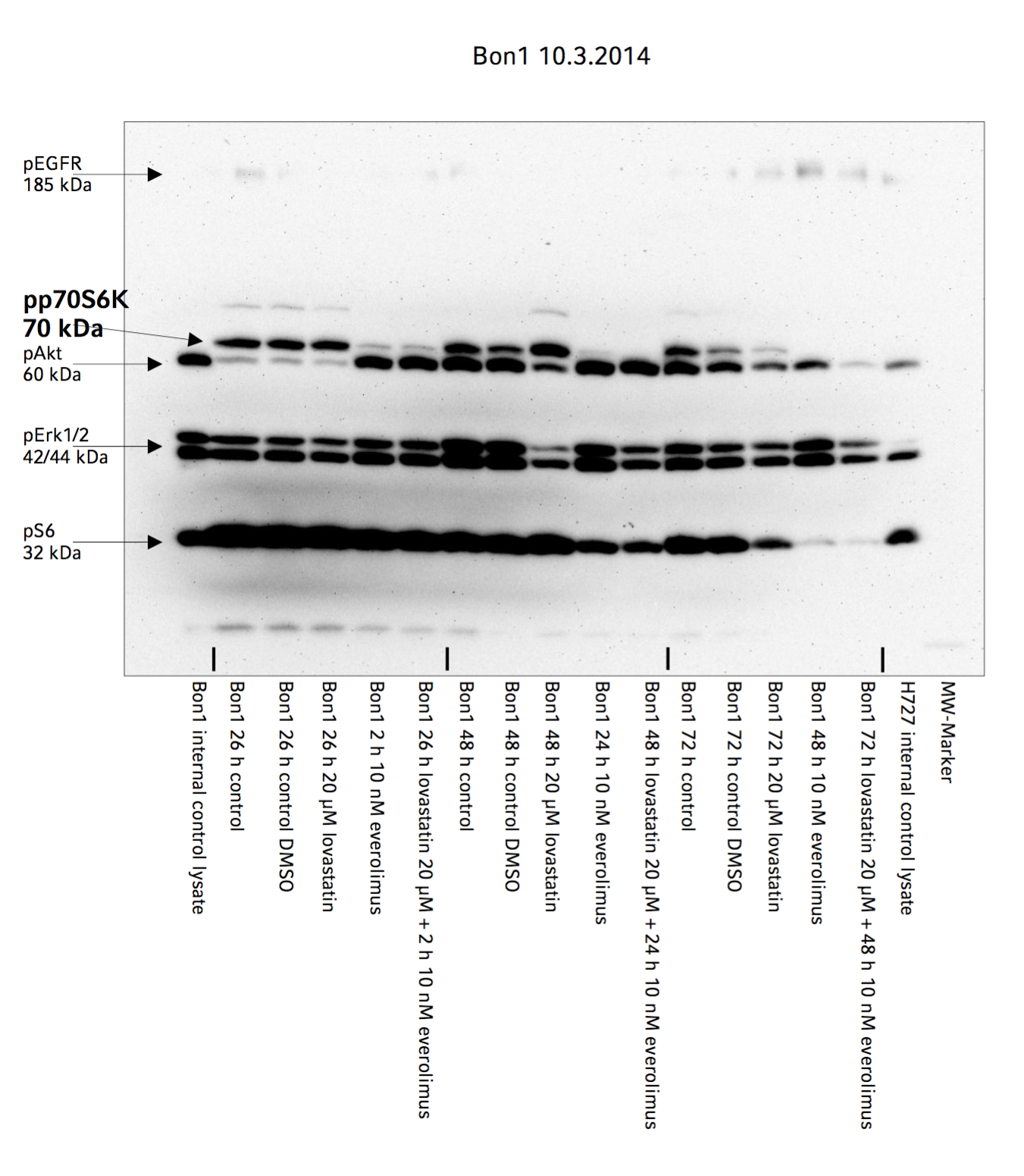

Supplement: S9 Fig — (TIF) [file pone.0143830.s009.tif]

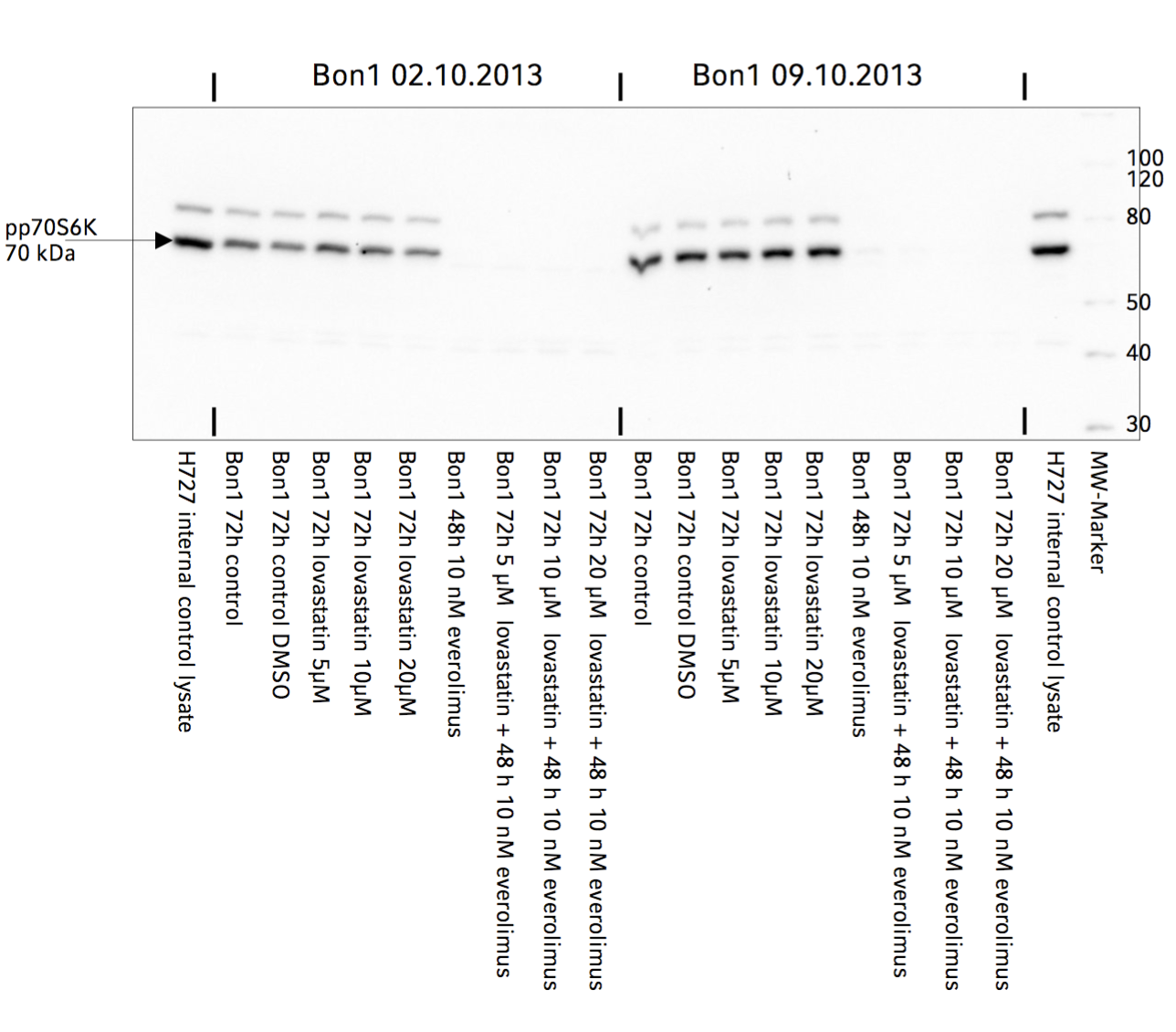

Supplement: S10 Fig — (TIF) [file pone.0143830.s010.tif]

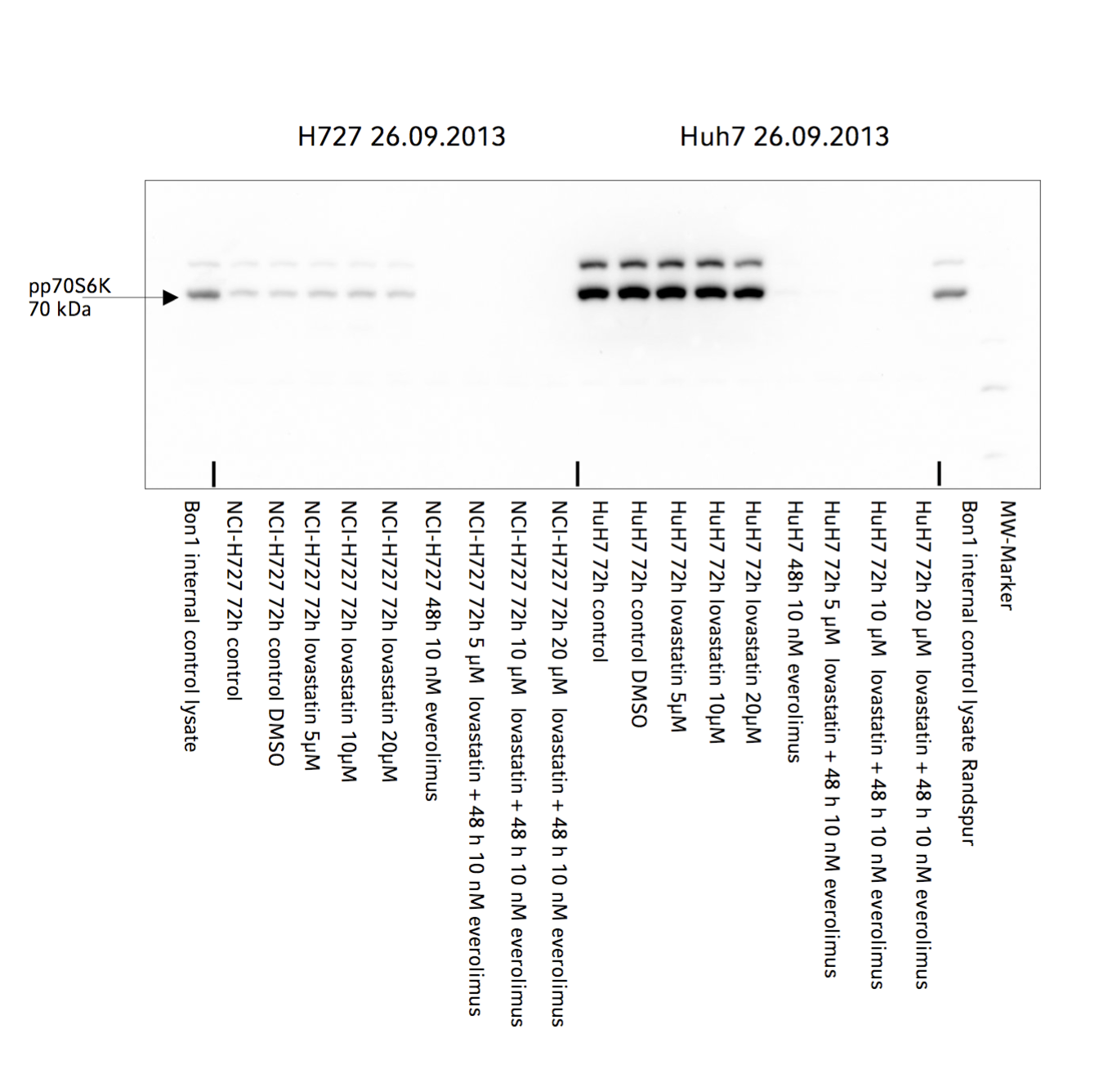

Supplement: S11 Fig — (TIF) [file pone.0143830.s011.tif]

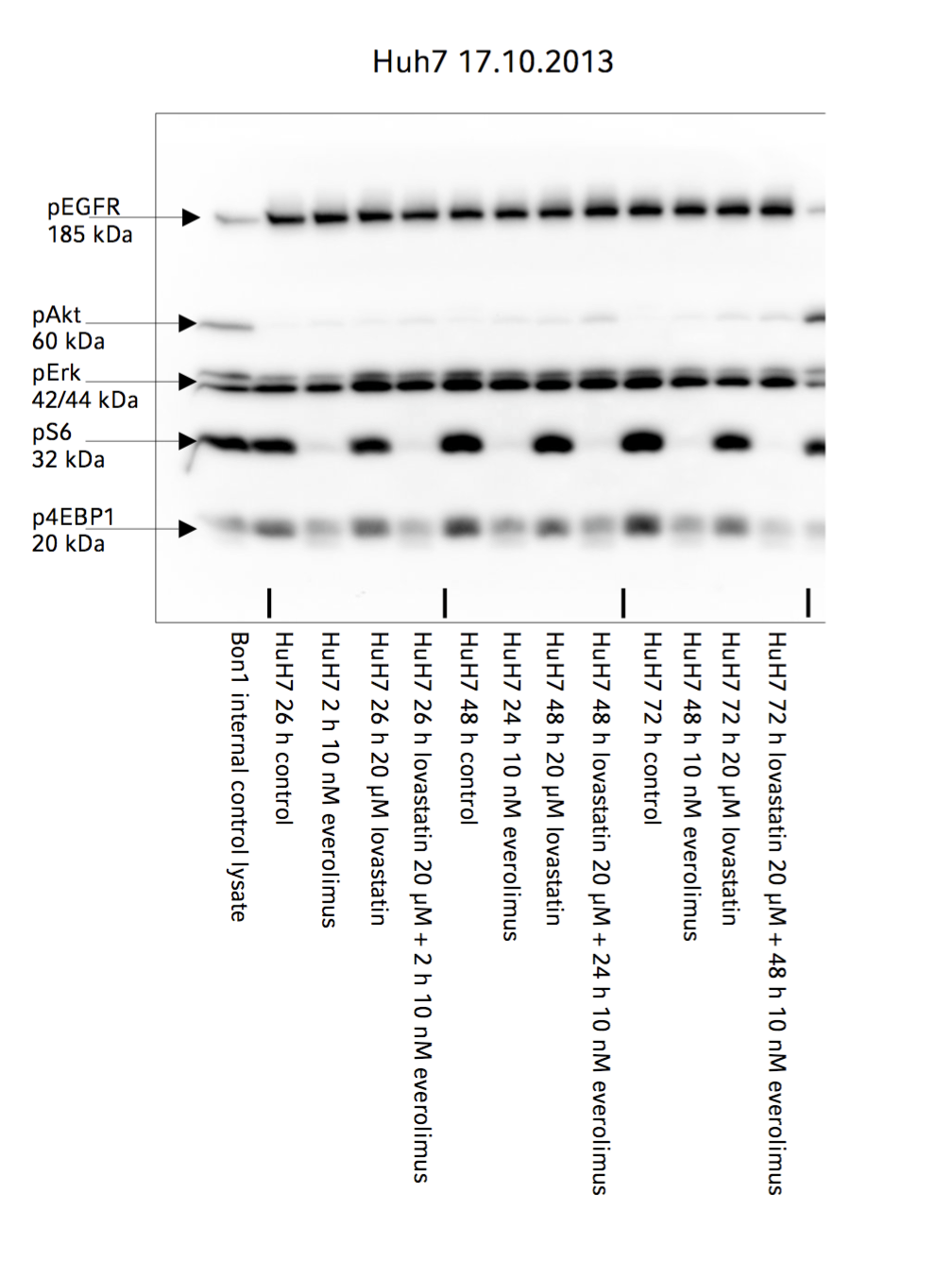

Supplement: S12 Fig — (TIF) [file pone.0143830.s012.tif]

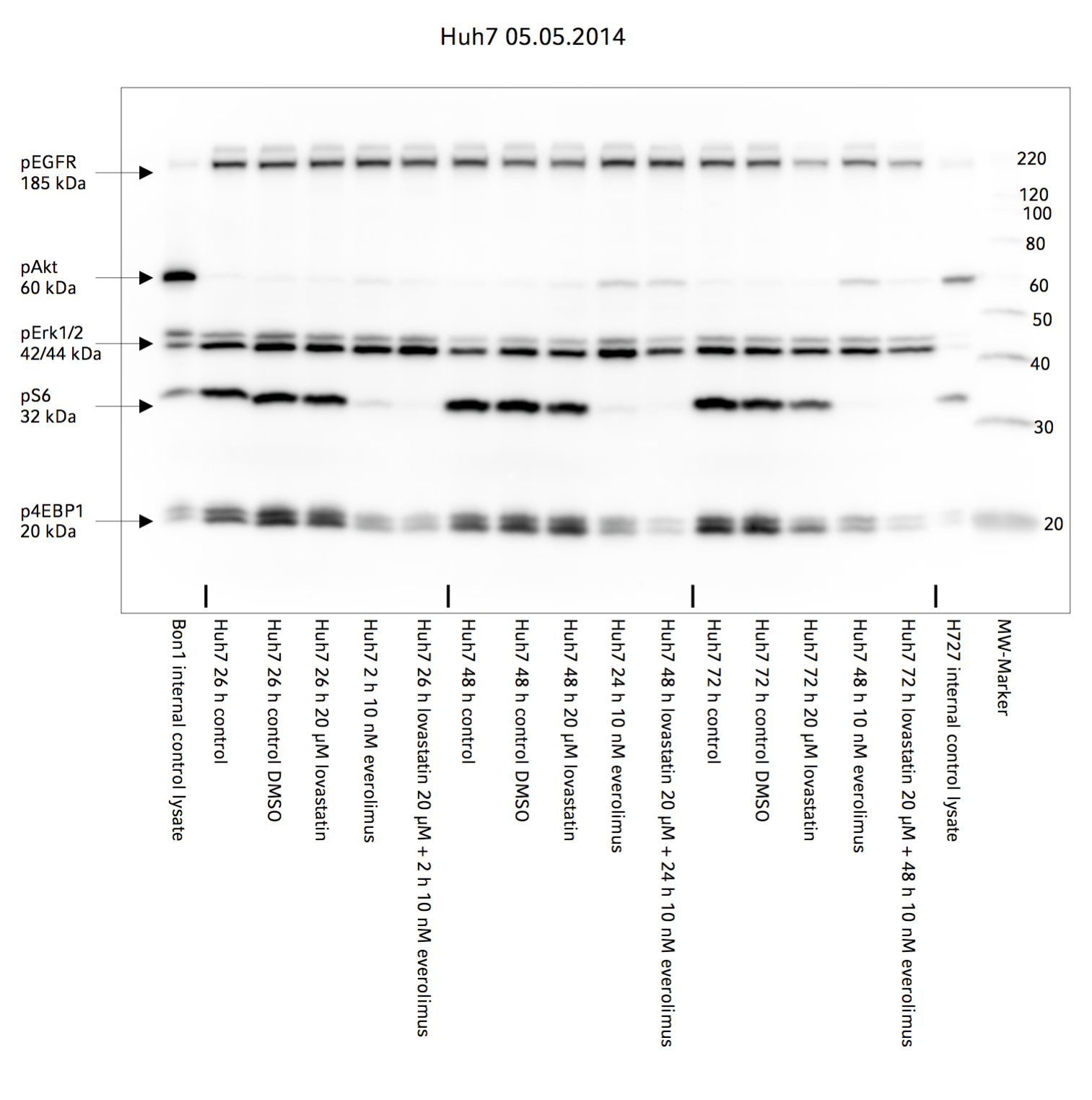

Supplement: S13 Fig — (TIF) [file pone.0143830.s013.tif]

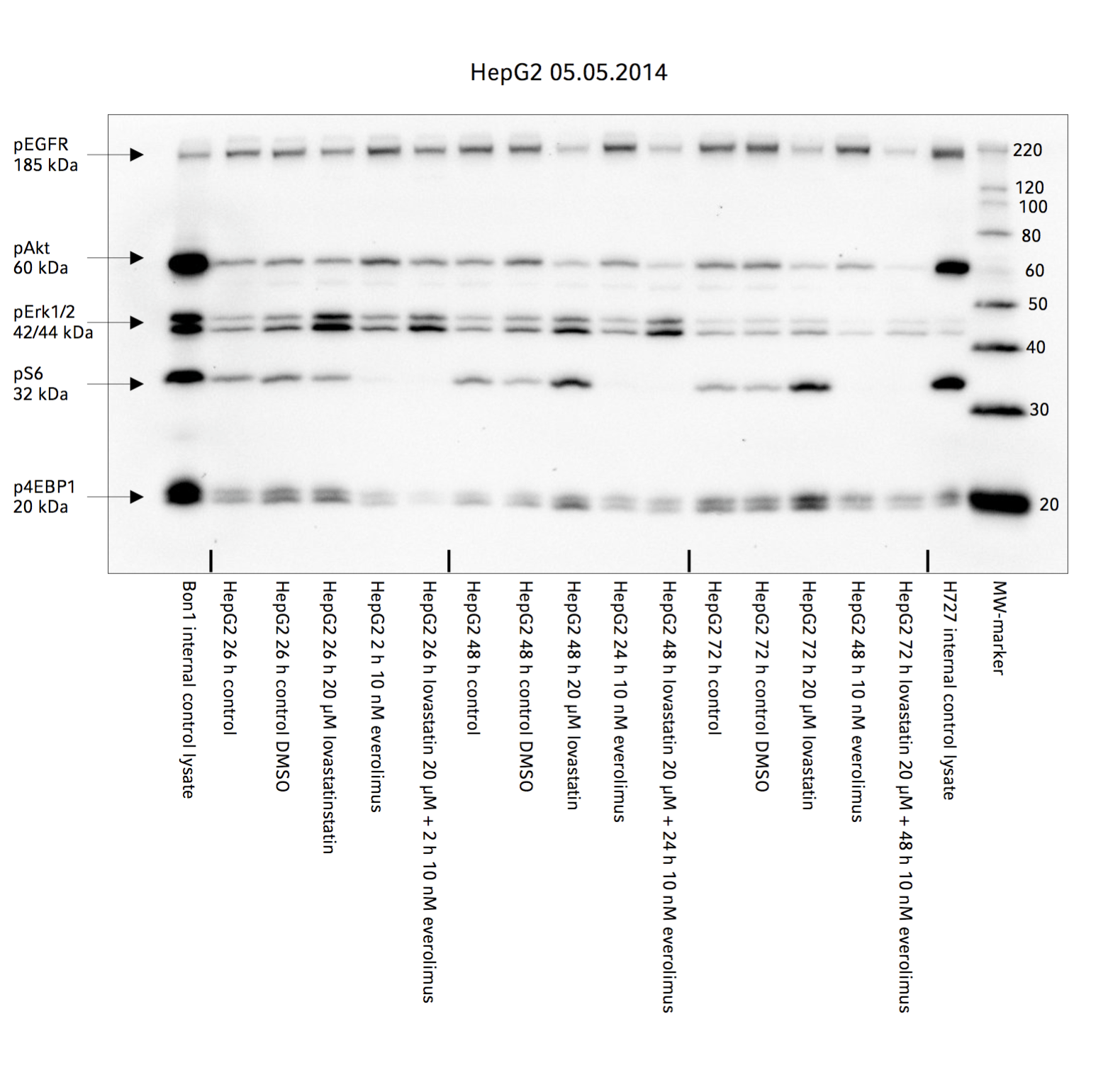

Supplement: S14 Fig — (TIF) [file pone.0143830.s014.tif]

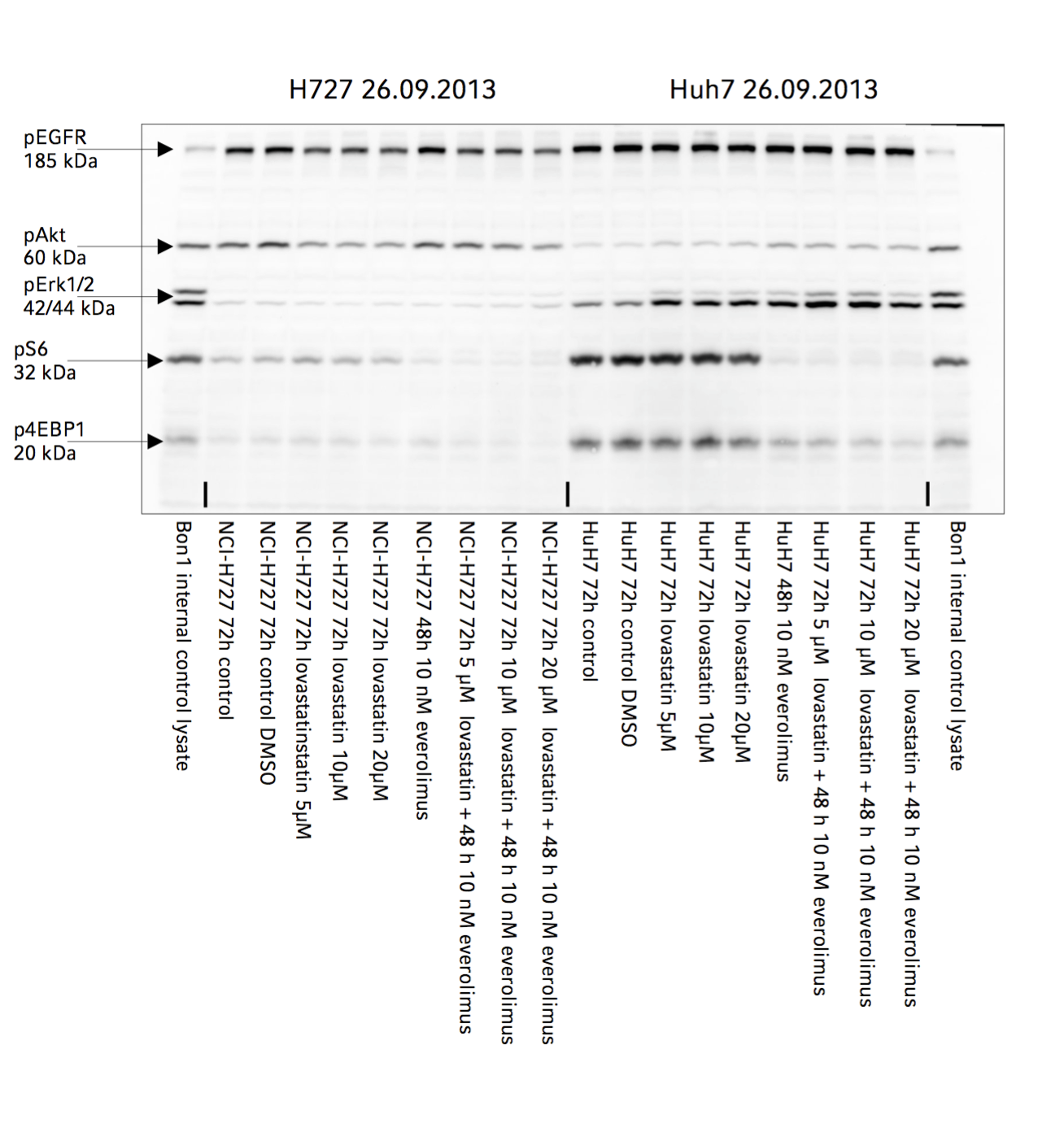

Supplement: S15 Fig — (TIF) [file pone.0143830.s015.tif]

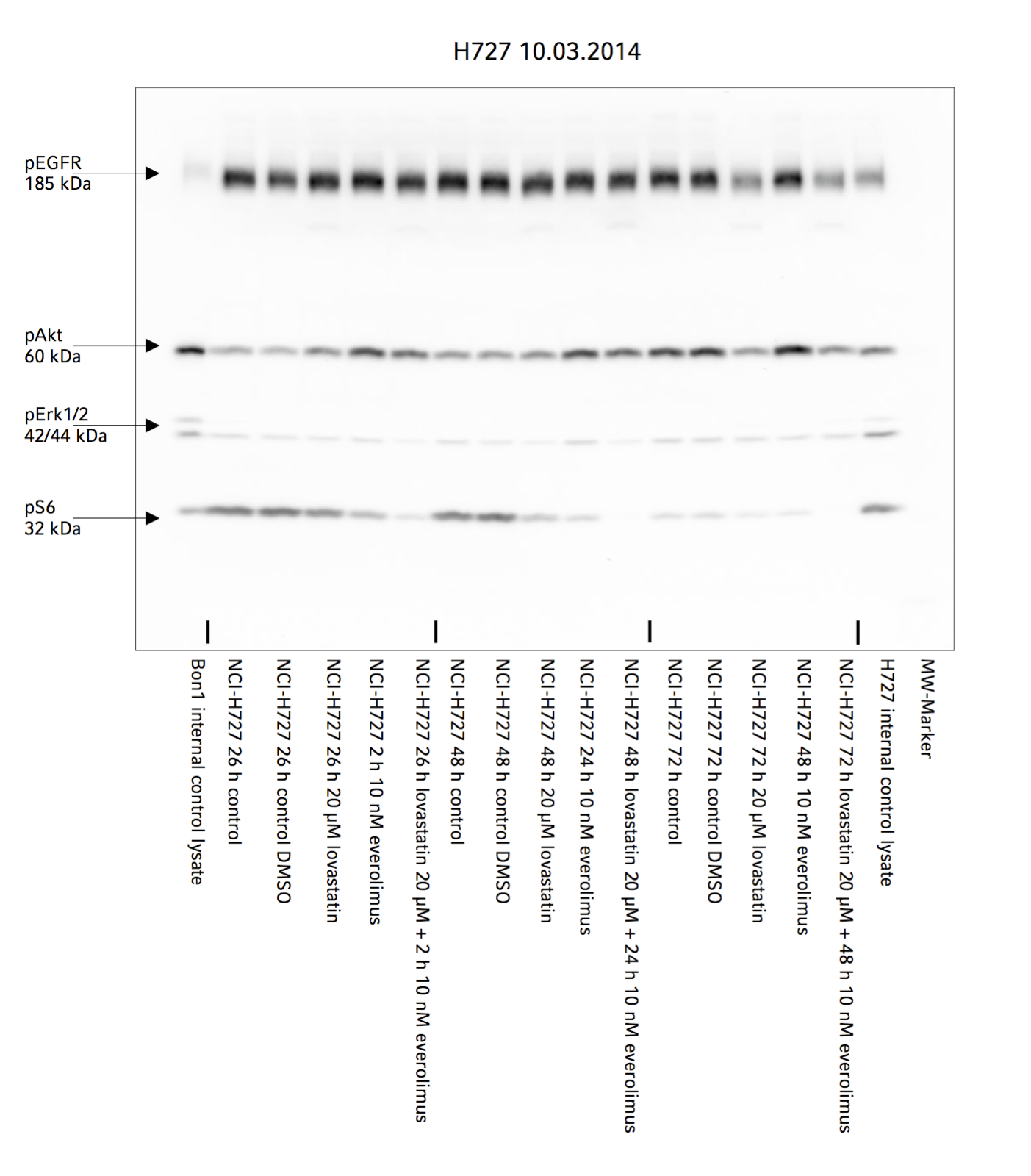

Supplement: S16 Fig — (TIF) [file pone.0143830.s016.tif]

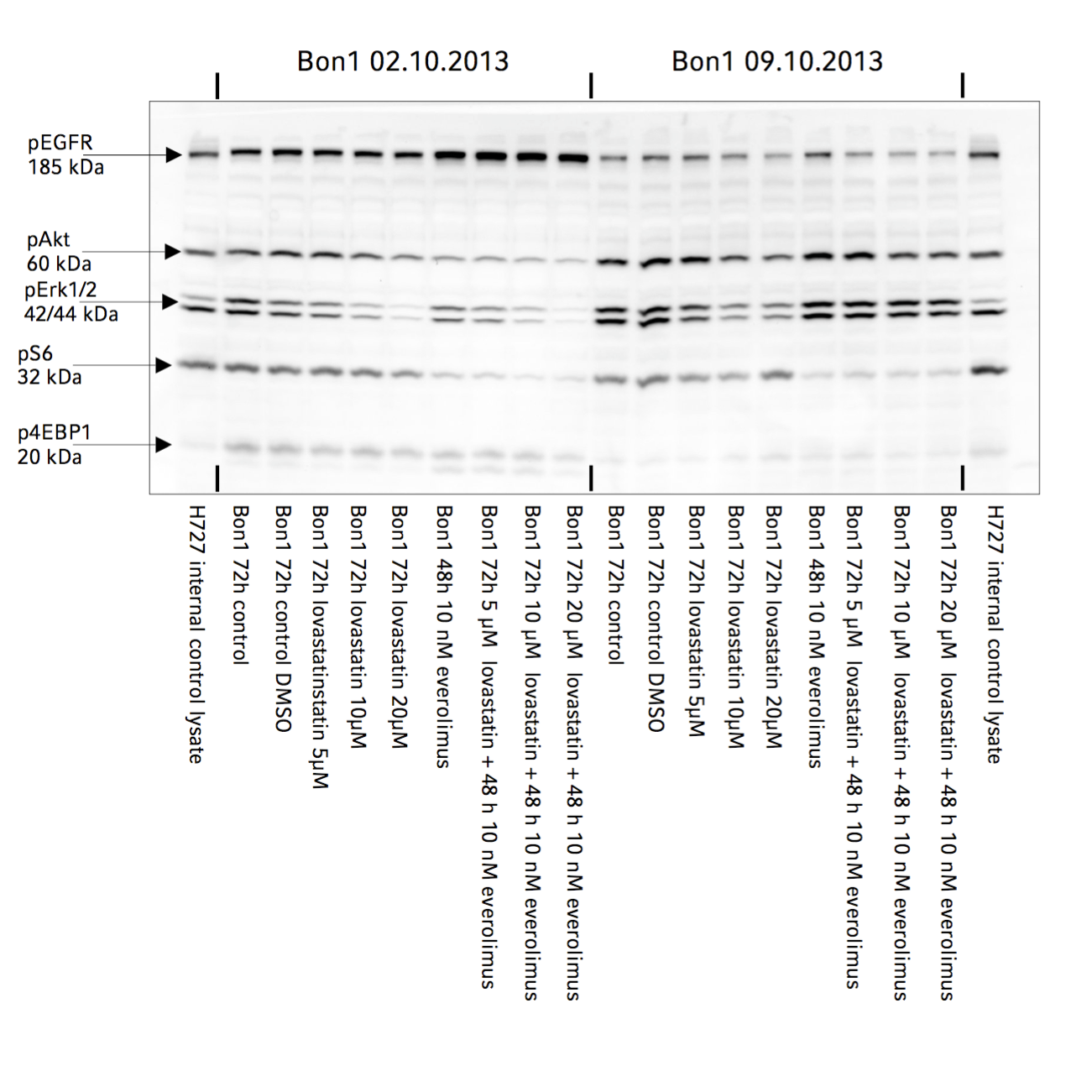

Supplement: S17 Fig — (TIF) [file pone.0143830.s017.tif]

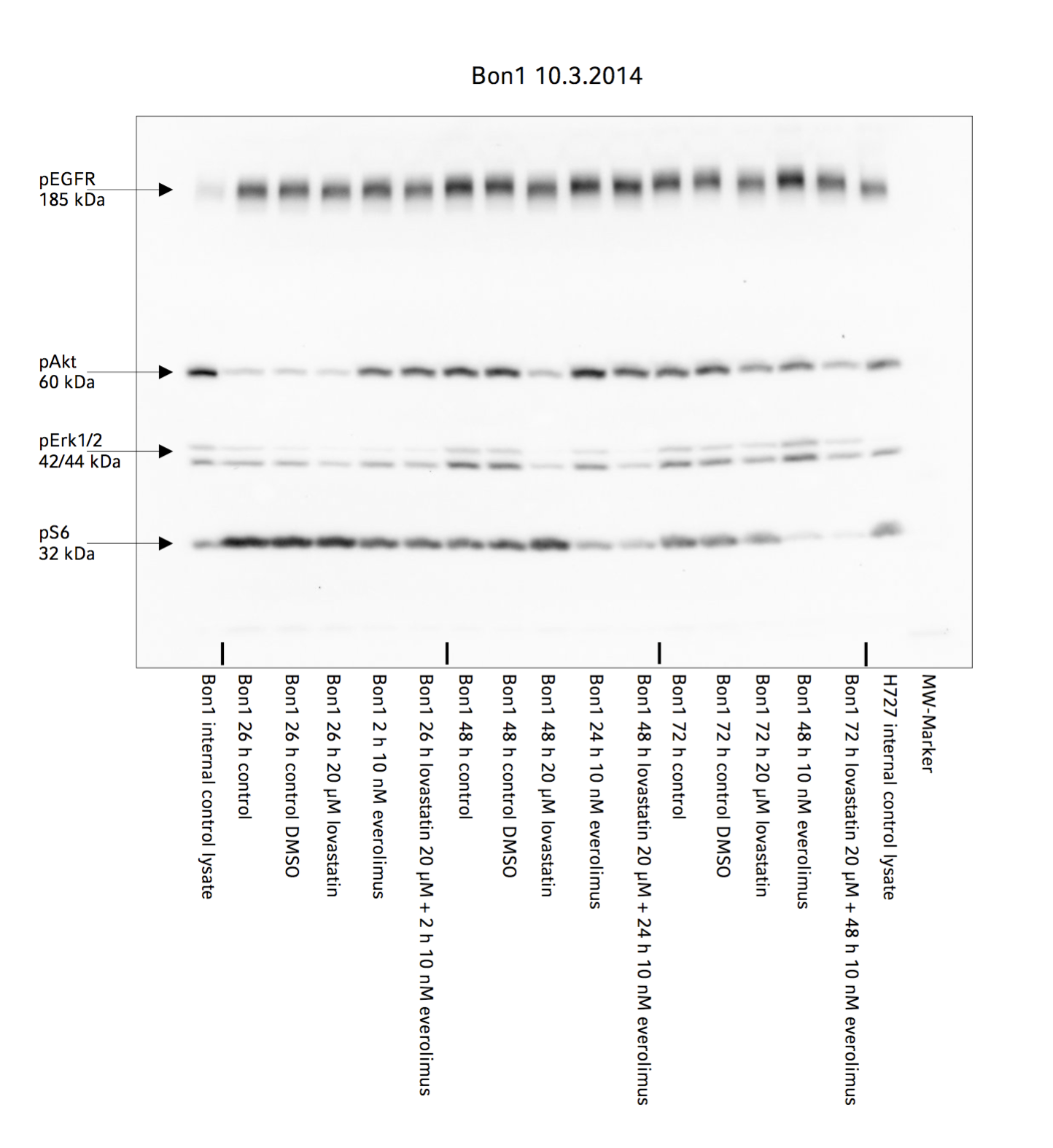

Supplement: S18 Fig — (TIF) [file pone.0143830.s018.tif]

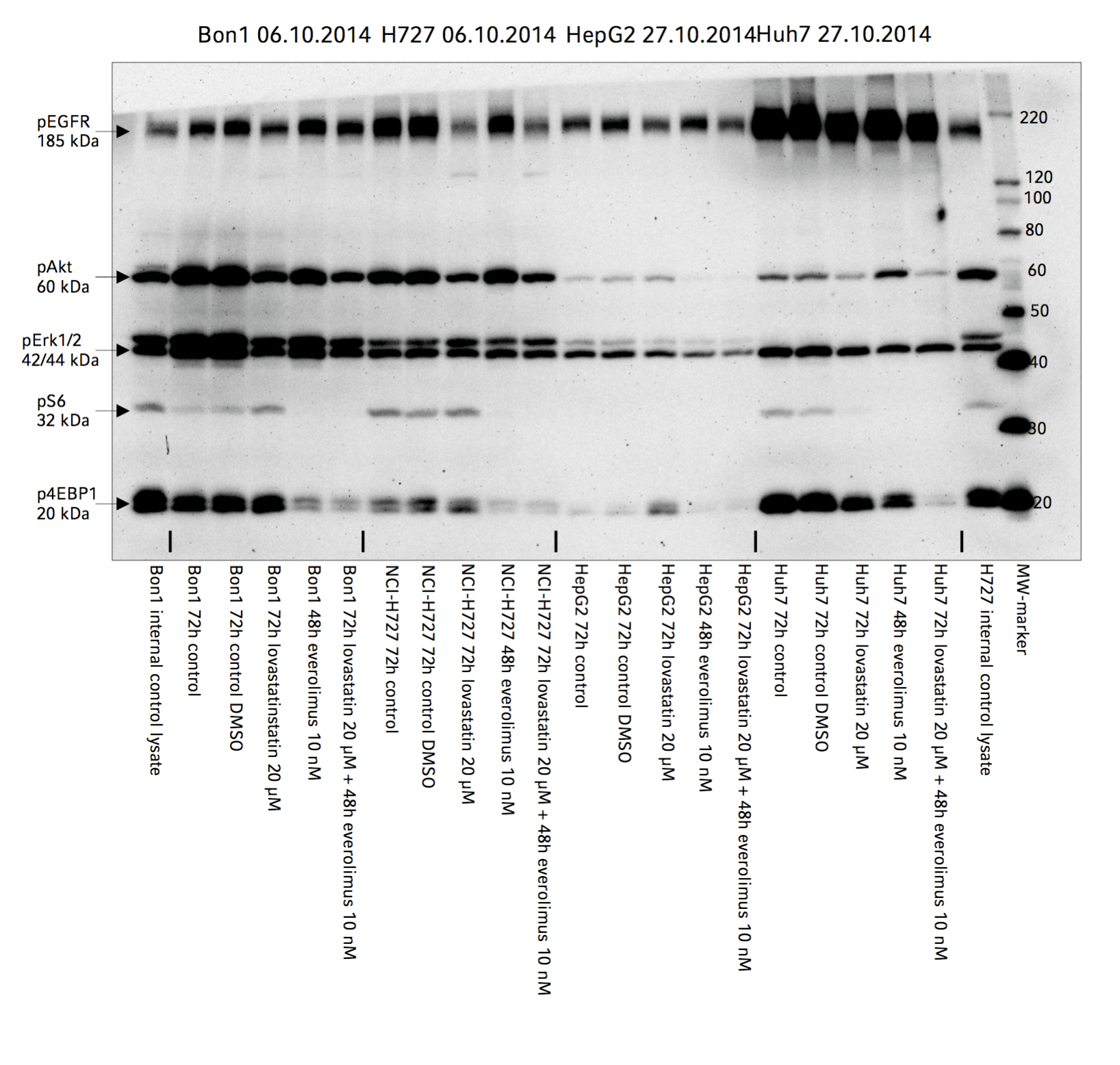

Supplement: S19 Fig — (TIF) [file pone.0143830.s019.tif]

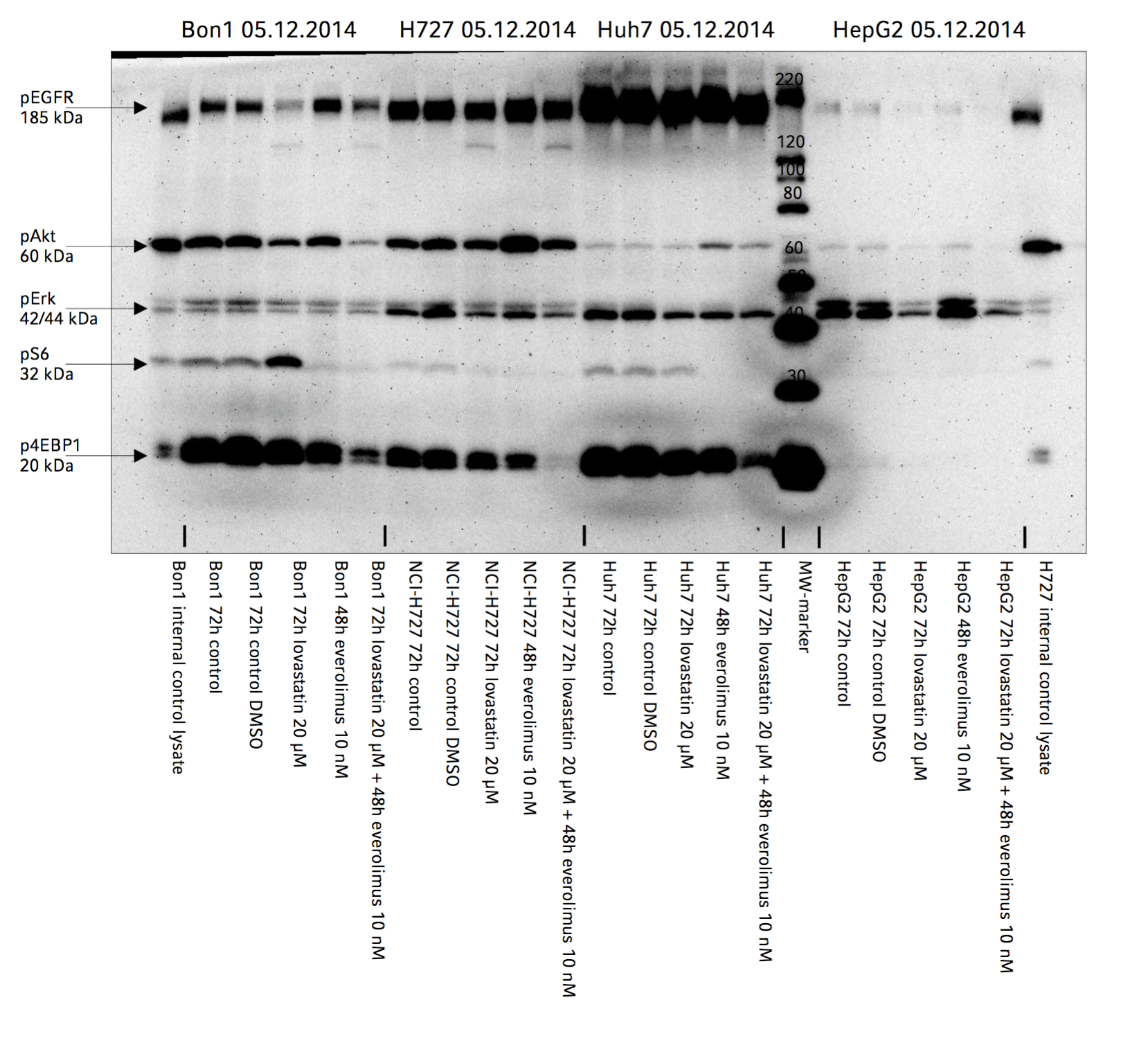

Supplement: S20 Fig — (TIF) [file pone.0143830.s020.tif]

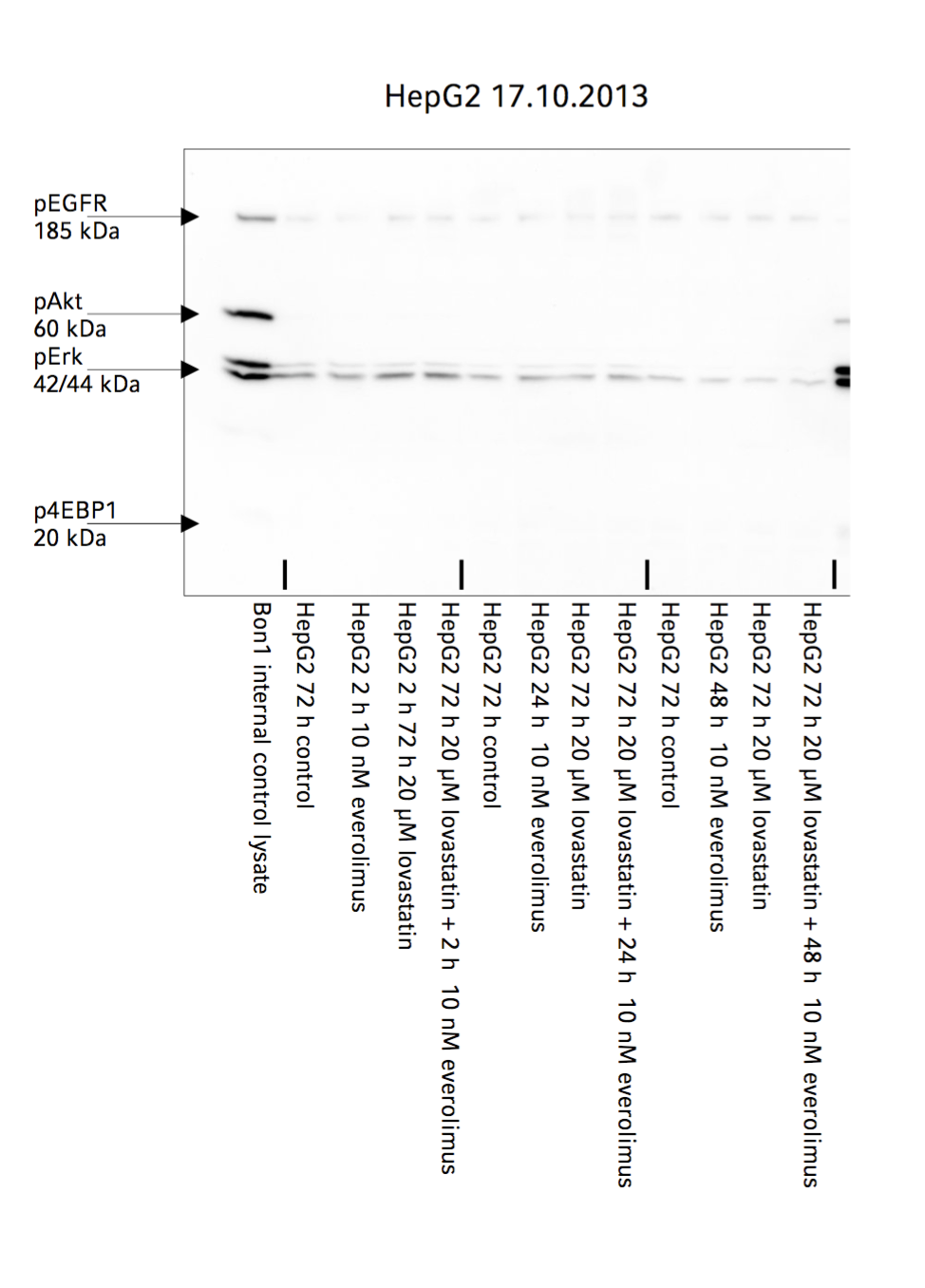

Supplement: S21 Fig — (TIF) [file pone.0143830.s021.tif]

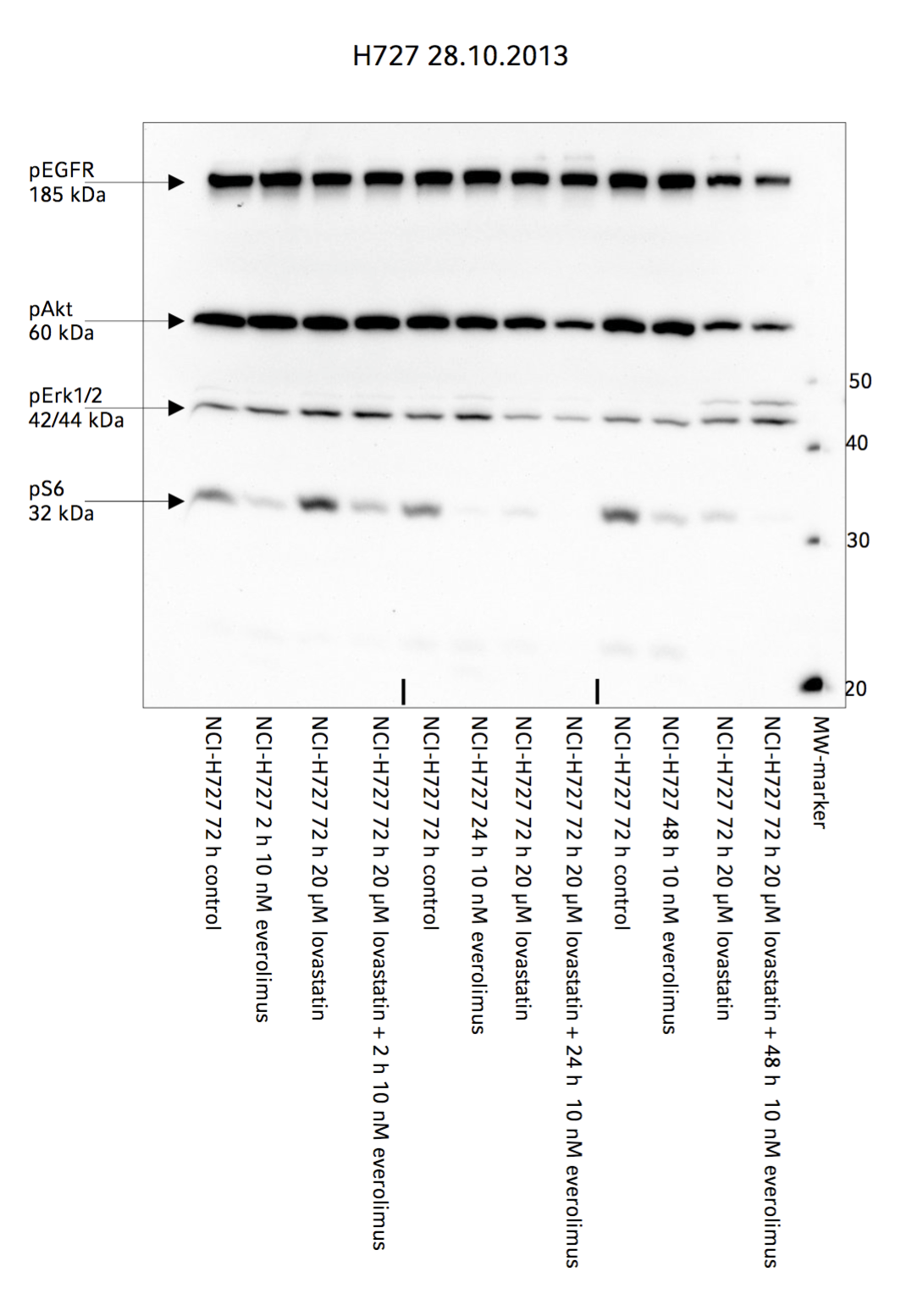

Supplement: S22 Fig — (TIF) [file pone.0143830.s022.tif]
